# Ingression Progression Complexes Control Extracellular Matrix Remodelling during Cytokinesis in Budding Yeast

**DOI:** 10.1371/journal.pgen.1005864

**Published:** 2016-02-18

**Authors:** Magdalena Foltman, Iago Molist, Irene Arcones, Carlos Sacristan, Yasmina Filali-Mouncef, Cesar Roncero, Alberto Sanchez-Diaz

**Affiliations:** 1 Instituto de Biomedicina y Biotecnología de Cantabria, Universidad de Cantabria, CSIC, Santander, Spain; 2 Departamento de Biología Molecular, Facultad de Medicina, Universidad de Cantabria, Santander, Spain; 3 Instituto de Biología Funcional y Genómica, Departamento de Microbiología y Genética, CSIC, Universidad de Salamanca, Salamanca, Spain; Howard Hughes Medical Institute and Vanderbilt University School of Medicine, UNITED STATES

## Abstract

Eukaryotic cells must coordinate contraction of the actomyosin ring at the division site together with ingression of the plasma membrane and remodelling of the extracellular matrix (ECM) to support cytokinesis, but the underlying mechanisms are still poorly understood. In eukaryotes, glycosyltransferases that synthesise ECM polysaccharides are emerging as key factors during cytokinesis. The budding yeast chitin synthase Chs2 makes the primary septum, a special layer of the ECM, which is an essential process during cell division. Here we isolated a group of actomyosin ring components that form complexes together with Chs2 at the cleavage site at the end of the cell cycle, which we named ‘ingression progression complexes’ (IPCs). In addition to type II myosin, the IQGAP protein Iqg1 and Chs2, IPCs contain the F-BAR protein Hof1, and the cytokinesis regulators Inn1 and Cyk3. We describe the molecular mechanism by which chitin synthase is activated by direct association of the C2 domain of Inn1, and the transglutaminase-like domain of Cyk3, with the catalytic domain of Chs2. We used an experimental system to find a previously unanticipated role for the C-terminus of Inn1 in preventing the untimely activation of Chs2 at the cleavage site until Cyk3 releases the block on Chs2 activity during late mitosis. These findings support a model for the co-ordinated regulation of cell division in budding yeast, in which IPCs play a central role.

## Author Summary

Cytokinesis is the process by which a cell divides in two and occurs once cells have replicated and segregated their chromosomes. Eukaryotic cells assemble a molecular machine called the actomyosin ring that drives cytokinesis. Contraction of the actomyosin ring is coupled to ingression of the plasma membrane and extracellular matrix remodelling. In eukaryotes, glycosyltransferases that synthesise polysaccharides of the extracellular matrix are emerging as essential factors during cytokinesis. Defects associated with the function of those glycosyltransferases induce the failure of cell division, which promotes the formation of genetically unstable tetraploid cells. Budding yeast cells contain a glycosyltransferase called Chs2 that makes a special layer of extracellular matrix and is essential during cell division. Our findings provide new insights into the molecular mechanism by which the cytokinesis regulators Inn1 and Cyk3 finely regulate the activity of glycosyltransferase Chs2 at the end of mitosis. In addition we isolated a group of actomyosin ring components that form complexes together with Chs2 and Inn1 at the cleavage site, which we have named ‘ingression progression complexes’. These complexes coordinate the contraction of the actomyosin ring, ingression of the plasma membrane and extracellular matrix remodelling in a precise manner. Chs2 is indeed a key factor for coordinating these events. It appears that similar principles could apply to other eukaryotic species, such as fission yeast even if the identity of the relevant glycosyltransferase has changed over the evolution. Taking into account the conservation of the basic cytokinetic mechanisms future studies should try to determine whether a glycosyltransferase similar to Chs2 plays a key role during cytokinesis in human cells.

## Introduction

Eukaryotic cells divide their cytoplasm at the end of mitosis in a highly regulated process called cytokinesis, which safeguards inheritance of the genome and organelles by the two daughter cells. The failure of cell division results in the formation of genetically unstable tetraploid cells, which may give rise to cancer [1] [2]. The successful completion of cytokinesis requires the precise coordination between an actomyosin-based contractile ring, which drives the ingression of the plasma membrane, and the remodelling of the extracellular matrix (ECM) [3] [4] [5] [6]. Yeast cells are surrounded by rigid ECM known as the cell wall, which provides the structural support and protection necessary to survive as unicellular organisms. The ECM is composed of a collection of biochemically distinct components, among which polysaccharides are emerging as key factors during cytokinesis, as shown by the failure in cytokinesis caused by defects associated with their synthesis in evolutionary distant organisms such as the budding yeast *Saccharomyces cerevisiae* [7] [8], the fission yeast *Schizosaccharomyces pombe* [9] [10] [11], the nematode *Caenorhabditis elegans* [12] and the mouse [13]. In these four examples, the impairment of a glycosyltransferase determines clear cell division defects. In budding yeast, it is the glycosyltransferase chitin synthase II, a transmembrane protein encoded by *CHS2*, which centripetally produces a distinct layer of chitin between mother and daughter cells during cytokinesis, called the primary septum, which is essential for life [6]. Chitin is a polymer of N-acetylglucosamine (Glc-NAc), which is synthesised from an activated nucleotide substrate UDP-N-acetylglucosamine (UDP-GlcNAc), and chitin chains are subsequently secreted outside the cells, assembled into microfibrils and organised in the extracellular matrix [14] [15].

In yeast cells, primary septum formation is tightly coupled to actomyosin ring contraction and ingression of the plasma membrane at the cleavage site [6]. In fact, defects associated with one of those processes perturb the others, although the underlying mechanisms linking them together remain unclear [16] [17] [18] [8] [19] [20]. The primary septum, which is later flanked by secondary septa, is finally digested to allow separation of the two daughter cells [21].

The core components and mechanisms of cytokinesis are largely conserved from yeast to humans, which makes the budding yeast cells an attractive model for studying the process of eukaryotic cytokinesis and for identifying how cells coordinate such processes [22] [5] [6]. Successful cytokinesis requires mechanisms that timely and effectively orchestrate the completion of the different steps along the cell cycle. First, cells need to assemble a contractile ring containing type II myosin and many other factors at the cleavage site, in a sequential and highly regulated process. At the early stages of the cell cycle, the type II myosin Myo1 forms a ring at the place that will later become the division site [16] [23]. Myo1 plays a scaffolding role in the assembly of the cytokinetic machinery [24] and associates with other factors during mitosis. These include actin-nucleating and bundling factors such as formins and the IQGAP protein Iqg1, leading to the assembly of a functional contractile actomyosin ring at the end of anaphase [25] [26] [22] [5]. Iqg1 contains an amino terminal calponin homology domain, which is thought to crosslink actin filaments, followed by IQ repeats that interact with Hof1 [27]. Interestingly, Hof1 interacts directly with type II myosin Myo1 and localises at the cleavage site in a complex manner, which depends upon Myo1 [28] [29]. In addition, it has recently been described that Hof1 shares a role with Rvs167 in actin ring assembly and Iqg1 recruitment to the bud-neck [30]. Hof1 contains an F-BAR domain in its N-terminal region and an SH3 domain in its C-terminus, both of which have been shown to be important for dynamics and function of the Hof1 protein [28] [29]. The SH3 domain of Hof1 is known to interact with proline-rich motifs (PXXP) located at the C-terminus of Inn1 [19] [31] [30]. Cells depleted for Inn1 still allow contraction of the actomyosin ring, but membrane ingression fails and the primary septum is not formed, despite the presence of Chs2 [19] [31]. In addition to Hof1, Inn1 interacts with Iqg1 [19] and with Cyk3, through the SH3 domain of Cyk3 located at its N-terminus [32] [31] [33] [30]. Furthermore, Hof1 SH3 binds to a proline-rich stretch of Cyk3 [34]. Taken together, it seems that multiple actomyosin components share binary interactions, but until now there has been no evidence that they all interact together to form large complexes in cells, in order to perform coordinated functions during cytokinesis.

Following full assembly of the contractile ring, primary septum formation occurs when cells have segregated their chromosomes and actomyosin ring contraction initiates. The expression, localisation and enzymatic activity of chitin synthase Chs2 are temporally and spatially regulated [35] [36] [8] [37] [38] [39]. Recent findings suggest that Hof1, Inn1 and Cyk3 regulate chitin synthase during cytokinesis in budding yeast, although the molecular mechanism is poorly understood [19] [31] [32] [40] [20]. Hof1 interacts directly with Chs2 and stabilises the chitin synthase at the cleavage site [29]. It also appears that Cyk3 could regulate Chs2 activity, since an increased dosage of Cyk3 stimulates Chs2-dependent chitin synthesis and the formation of primary-septum-like structures at the bud neck [41] [42]. Moreover, we found genetic evidence that enhanced chitin synthase activity associated with a hypermorphic allele of *CHS2*, *CHS2-V377I*, suppresses the defects associated with an inactive form of the C2 domain of Inn1 (first 134 amino acids of Inn1) and deficiencies associated with the lack of Cyk3 in budding yeast cells [20].

Here, we have isolated complexes containing the actomyosin ring components Myo1, Iqg1, Hof1, Inn1 and Cyk3 all together with chitin synthase Chs2 from cells undergoing cytokinesis, which we named ‘ingression progression complexes’ or IPCs. We show that IPCs are assembled at the end of the cell cycle and we propose that IPCs coordinate contraction of the actomyosin ring, plasma membrane ingression and primary septum deposition in budding yeast. We find that IPC components co-operate to recruit Chs2 to the division site. Moreover, we provide evidence that Inn1 and Cyk3 interact directly with the catalytic domain of Chs2. Our data indicate that the C2 domain of Inn1 and the transglutaminase-like domain of Cyk3 increase the chitin synthase activity associated with Chs2. We used an experimental system to find a previously unanticipated role for the C-terminus of Inn1 in preventing the untimely activation of Chs2 at the cleavage site until Cyk3 releases the block on Chs2 activity, when cells reach the end of the cell cycle

## Results

### Inn1 binds directly to the catalytic domain of Chs2

**Fig 1:**
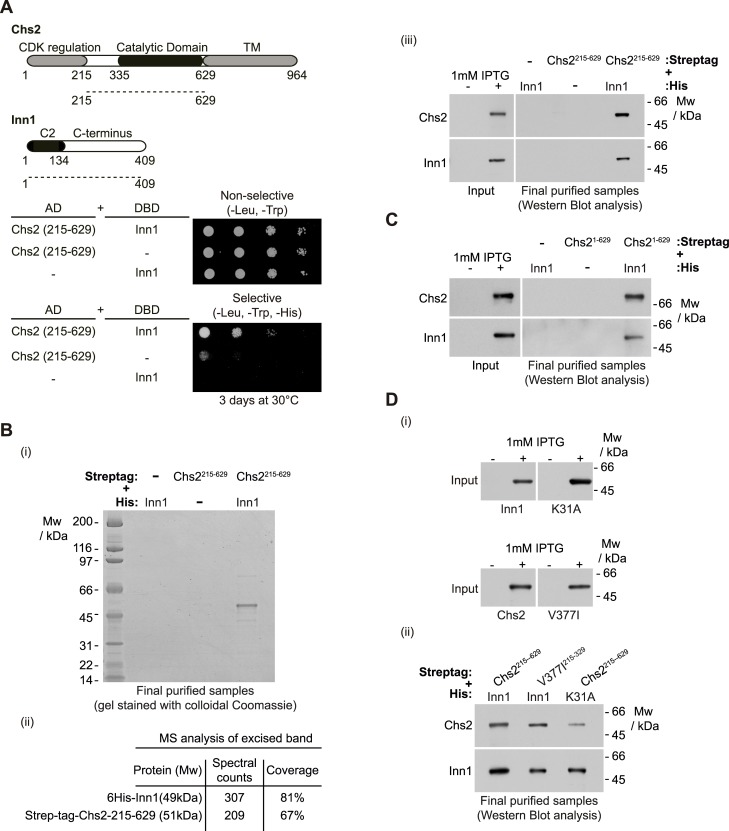
Inn1 interacts directly with the catalytic domain of Chs2. **(A)** Truncated allele of Chs2 containing the catalytic domain and Inn1 were used to show that this region of Chs2 (Chs2-215-629) interacts in a yeast two-hybrid assay with Inn1. **(B)** Chs2-215-629 interacts directly with full-length Inn1. After induction with IPTG, pairs of *E*. *coli* cell cultures expressing the indicated recombinant protein fragments were mixed and used to purify putative protein complexes. The final purified fractions were analysed by SDS-PAGE and the gels were stained with colloidal Coomassie (i). Both Inn1 and Chs2-215-629 migrate similarly in SDS-PAGE gels (6His-Inn1(49kDa) and Strep-tag-Chs2-215-629 (51kDa)). The acrylamide band was cut out from the gel and protein composition, spectral counts and coverage were analysed by mass spectrometry (ii). The final purified fractions from (i) were analysed by immunoblotting and the tagged proteins were detected with anti-Streptag or anti-His antibodies (iii). **(C)** Chs2-1-629 interacts directly with full-length Inn1. Pairs of *E*. *coli* cell cultures expressing the indicated protein fragments were mixed and used to purify and analyse putative protein complexes as in (B). **(D)** After induction with IPTG (i) pairs of *E*. *coli* cell cultures expressing the indicated protein fragments were mixed and used to purify and analyse putative protein complexes as in (B) (ii).

We previously found that Inn1 co-purified with Chs2 when studying cells that had been released into mitosis from a G2-M block to allow them to undergo cytokinesis synchronously [20]. To study further the interaction between Inn1 and Chs2, we used several approaches. First, we used the yeast two-hybrid assay to show that a fragment of Chs2 that contains its catalytic domain (Chs2-215-629) was able to interact with full-length Inn1 (Fig 1A). We then determined whether these factors were able to interact directly in an extract of *E*. *coli* cells. We generated an *E*. *coli* strain that expressed 6His-tagged Inn1 and, in parallel, another strain that expressed a truncated version of Chs2 fused to Streptag (Streptag-Chs2-215-629), as indicated in S1A Fig. We then mixed the cultures and generated a single cell extract containing Inn1, Chs2 and all the native *E*. *coli* proteins (S1A Fig). We initially purified the truncated version of Chs2 from the cell extracts, and subsequently isolated 6His-Inn1 from the purified material. In this way, we found that Chs2 co-purified specifically with Inn1 (Fig 1B). Note that both Inn1 and Chs2-215-629 migrate similarly in SDS-PAGE gels, and so their presence was confirmed by mass spectrometry and immunoblotting analysis (Fig 1Bii and 1Biii). Following the same purification procedure described above, we found that a fragment of Chs2 that contains its CDK-regulated N-terminal domain together with its catalytic domain (Chs2-1-629, which only lacks the transmembrane domain) co-purified specifically with Inn1 (Fig 1C). Furthermore, we determined that formation of Chs2-Inn1 complex was not abolished by the Inn1-K31A mutation, which disrupts the function of the Inn1 C2 domain, or by a hypermorphic mutation in the catalytic domain of Chs2 (Chs2-V377I), which enhances its activity *in vitro* (Fig 1D).

### The C2 domain of Inn1 directly binds to and regulates the catalytic activity of Chs2

**Fig 2:**
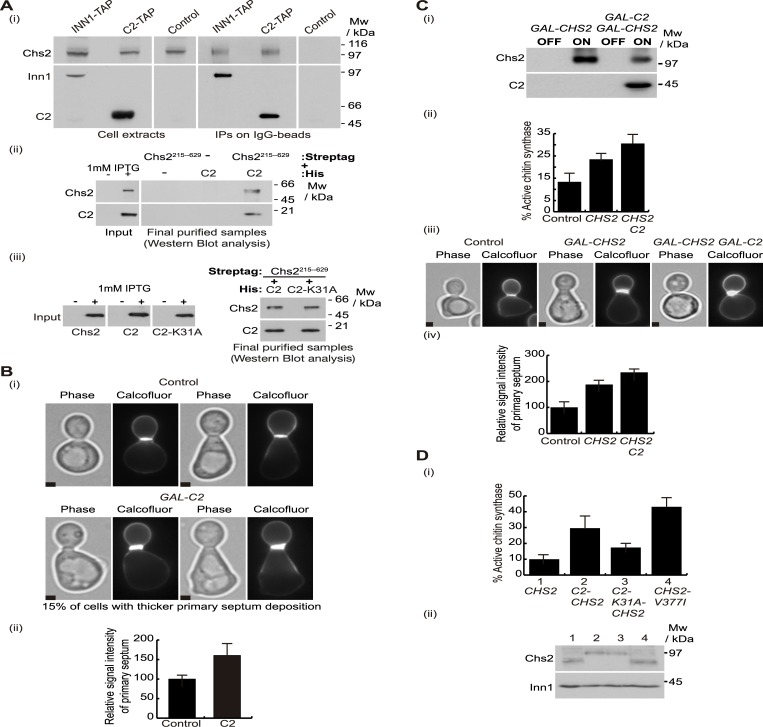
C2 domain of Inn1 directly binds to and regulates the catalytic activity of Chs2. **(A)** *INN1-TAP CHS2-9MYC* (YMF38), *C2-TAP CHS2-9MYC* (YMF88) and control (YAD382) strains were grown at 24°C in YPD medium, arrested in G1 phase by the addition of alpha factor, and then released in YPD medium for 105 minutes. Cell extracts were made and Inn1-TAP or C2-TAP were immunoprecipitated on IgG beads before detection of the indicated proteins by immunoblotting (i). After induction with IPTG, pairs of *E*. *coli* cultures expressing 6His-tagged-Inn1-C2 and Strep-tag-Chs2-215-629 were mixed and used to purify putative protein complexes following scheme in S1A (see Materials and Methods). The final purified fractions were analysed by SDS-PAGE and tagged proteins were detected with anti-Streptag or anti-His antibodies (ii). Pairs of *E*. *coli* cultures expressing 6His-tagged-Inn1-C2 or 6His-tagged-Inn1-C2-K31A were mixed with Strep-tag-Chs2-215-629 and used to purify putative protein complexes as in (ii). The final purified fractions were analysed by SDS-PAGE and tagged proteins were detected with anti-Streptag or anti-His antibodies (iii). **(B)** *chs3Δ* control (YMF505) and *GAL-C2 chs3Δ* (YRK3) cells were grown in YPRaff medium at 24°C and synchronised in G1 with alpha factor. Subsequently, cells were released in YPGal for 135 minutes from G1 block in the presence of calcofluor to visualise primary septum deposition. 100 cells with primary septum for each sample were examined and we found that 15% of *GAL-C2 chs3Δ* cells had clearly higher intensity at the primary septum than the average intensity in control cells. Examples of these cells are shown in (i). Scale bars correspond to 2μm. The relative signal intensity of primary septum was measured for 100 cells and compared to control cells, where signal intensity was set to 100% (ii). **(C)** C2 domain of Inn1 increases the catalytic activity of Chs2. The protein levels of overexpressed Chs2 and C2 proteins (i) and percentage of active chitin synthase (ii) in *chs3Δ* control cells and cells lacking Chs3 and overexpressing either *GAL-CHS2* (YMF687) or *GAL-CHS2 GAL-C2* (YMF581) were determined in membranes isolated from asynchronous cultures (see Materials and Methods). Control, *GAL-CHS2* (YMF687) and *GAL-CHS2 GAL-C2* (YMF581) were grown as in (B) and stained with calcofluor to visualise primary septum deposition. 100 cells with primary septum for each sample were examined and examples of these cells are shown in (iii) and the relative signal intensity of primary septum was measured and compared to control cells, where signal intensity was set to 100% (iv). Scale bars correspond to 2μm. **(D)** The chitin synthase activity in *chs3Δ* cells expressing *CHS2* (YMF191), *C2-CHS2* (YMF172), *C2-K31A-CHS2* (YMF174) or *CHS2-V377I* (YMF192) was determined as in (C) (i). Cells were grown in YPD containing 0.1mM CuSO_4_ since *CHS2* and *CHS2* fusions were under the control of the *CUP1* promoter and protein expression levels of Chs2 and its fusions were determined (ii). Note that *CHS2* is highly expressed in (C), under the control of the *GAL1-10* promoter, whereas *CHS2* levels are much reduced in (D), under the *CUP1* promoter control.

Since it has been shown that inactivation of the C2 domain of Inn1 can be rescued by specific mutations in the catalytic domain of Chs2 that increase its activity [20], we tested whether the C2 domain of Inn1 directly associates with and regulates chitin synthase Chs2 *in vivo*. A yeast strain was generated in which either wild-type Inn1 or the Inn1 C2 domain were fused to the tandem affinity purification (TAP) tag and expressed under the control of the *INN1* promoter. *INN1-TAP*, *C2-TAP* and control strains were grown at 24°C, synchronised in G1 phase of the cell cycle by the addition of mating pheromone and cells were then released from G1 arrest. The resultant fusion proteins were isolated from cells going through cytokinesis synchronously 105 minutes after the release from G1 block, when localisation of Inn1 and Chs2 at the site of division peaks. We found that Chs2 co-purified specifically with the C2 domain of Inn1, equivalent to full-length Inn1 (Fig 2Ai). To test whether the C2 domain of Inn1 could interact directly with Chs2, we generated *E*.*coli* strains that produced 6His-tagged-Inn1-C2 and Strep-tag-Chs2-215-629 and proceeded as above. We found that 6His-C2 co-purified over two purification steps with Strep-tag-Chs2-215-629 (Fig 2Aii), indicating that the Inn1 C2 domain interacts directly with a fragment of Chs2 that contains its catalytic domain. In addition, we determined that interaction between Chs2 and the Inn1 C2 domain was not disrupted by Inn1-K31A mutation (Fig 2Aiii).

To investigate whether the Inn1 C2 could induce chitin synthase activity *in vivo*, we monitored the chitin level at the division site by calcofluor staining [43] in cells that overexpressed the C2 domain and lacked Chs3 (studies focused on Chs2 activity require the use of *chs3Δ* cells, because Chs3 is responsible for the synthesis of the vast majority of the chitin content in budding yeast cells [44]). Asynchronous cultures were grown at 24°C and cells were synchronised in G1 phase with mating pheromone. Subsequently, we released cells from G1 block into medium containing calcofluor to stain primary septa and galactose to allow overexpression of the C2 domain. Progression through cytokinesis and localisation of Chs2 at the site of division were similar in both control and *GAL-C2* cells (S1B Fig). To observe calcofluor-stained chitin in cells completing mitosis, cells were collected 135 minutes after release from G1 block (Fig 2B) when the percentage of cells containing primary septa peaks. We found that the signal intensity associated with calcofluor-stained chitin at the division site was higher in cells overexpressing C2 as compared to control cells (Fig 2B), indicating that the C2 domain of Inn1 is able to induce septum formation.

To test whether the C2 of Inn1 positively regulates the chitin synthase Chs2, two different *in vitro* approaches were used. First, two *chs3Δ* yeast strains were generated to overexpress either *CHS2* or *CHS2* together with the C2 domain. We grew control, *GAL-CHS2* and *GAL-CHS2 GAL-*C2 strains asynchronously in the presence of raffinose and then switched to medium containing galactose to induce the expression of Chs2 and the C2 domain (Fig 2Ci). After two hours we isolated membranes to perform a chitin assay, as previously reported [45] [20]. We found that the Inn1 C2 domain had an effect on Chs2 activity, since overexpression of C2 at the same time as Chs2 caused a 30% increase in the percentage of active chitin synthase (Fig 2Cii, compare *CHS2* with *CHS2 C2*). Consistently, we observed that cells overproducing the C2 domain and Chs2 induced thicker primary septum deposition (Fig 2Ciii and 2Civ).

Second, we fused the Inn1 C2 domain to Chs2 and measured the enzymatic activity associated with the C2-Chs2 fusion protein. We have previously shown that *C2-CHS2* fusion fully supports cytokinesis in *inn1Δ* cells [20]. We grew cells asynchronously, isolated membranes and performed an *in vitro* chitin assay. Subsequently, we calculated the percentage of active chitin synthase associated with C2-Chs2 and found that it increased 3-fold in comparison with Chs2 activity in control cells (Fig 2D, compare 1–2). This increase was significantly reduced when an inactive version of the C2 (C2-K31A) was fused to Chs2 (Fig 2D, compare 1–3). Taken together, these findings show that the Inn1 C2 domain directly binds to and regulates the catalytic domain of Chs2, which is required to form the primary septum during cytokinesis. Interestingly, the percentage of active chitin synthase associated with Chs2-V377I, increased 4.3 fold when compared with control Chs2 under the same conditions described above (Fig 2D(i), compare 1–4) while fusion proteins expression levels were similar (Fig 2D(ii)). This suggests that there might be other factors, in addition to the Inn1 C2, that contribute to Chs2 activation (Fig 2D, compare the difference between 1–2, 1–4 and 2–4).

### Systematic analysis of regulators of the chitin synthase Chs2 in budding yeast

**Fig 3:**
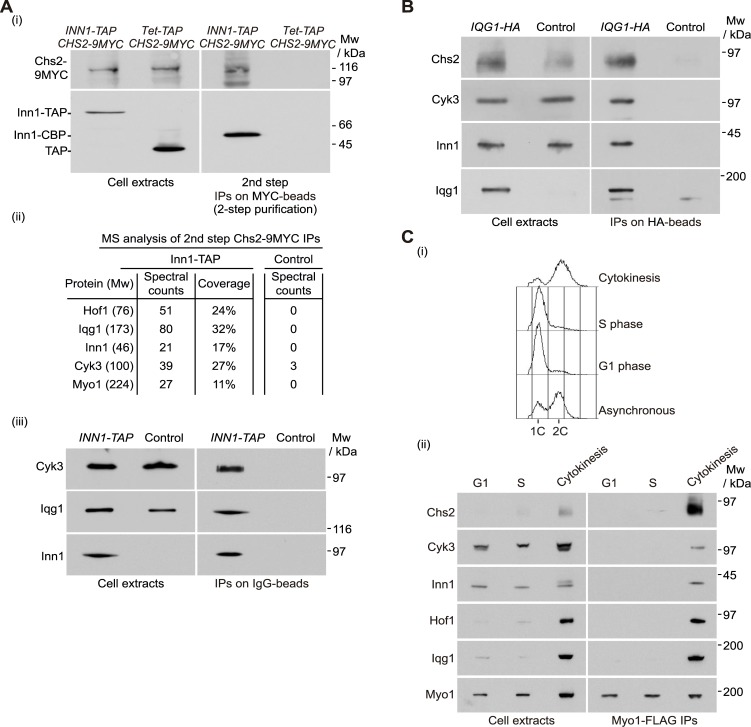
Identification of factors interacting with Chs2 and Inn1 during cytokinesis. **(A)** *INN1-TAP CHS2-9MYC* (YMF38) and control (YMF79) were grown at 24°C in YPD medium and synchronised in G1 phase with mating pheromone and then released for 105 minutes. Cell extracts were prepared before the immunoprecipitation of Inn1-TAP (or TAP in control) on IgG-beads. The isolated material was released from the beads by cleavage with TEV protease. After cleavage, part of the TAP tag (CBP, calmodulin-binding protein) remained fused to Inn1. Purified material was subjected to immunoprecipitation of Chs2-9MYC before analysis by SDS-PAGE and immunoblotting (i). The protein composition of purified fractions including spectral counts and coverage were analysed by mass spectrometry (ii). The *INN1-TAP IQG1-6HA* (YMF149) strain together with control (YMF152) were grown as in (i) and cell extracts were prepared and analysed by SDS-PAGE and immunoblotting (iii). **(B)** *IQG1-6HA* (YMF152) and control (YAD382) strains were grown as in (A) and cell extracts were prepared before the immunoprecipitation of Iqg1 and detection of the indicated proteins by immunoblotting. **(C)** *MYO1-FLAG* (YMF362) strain was grown at 24°C in YPD medium and synchronised in G1 phase with mating pheromone (G1 sample) or released from G1 block for 30 minutes (S-phase sample) or 105 minutes (cytokinesis sample). Cell cycle progression was monitored by flow cytometry (i). Cell extracts were then prepared before the immunoprecipitation of Myo1-FLAG on anti-FLAG-beads and detection of the indicated proteins by immunoblotting (ii).

To understand how cells control the activity of Chs2 at the division site during cytokinesis, we aimed to isolate Inn1-Chs2 complexes specifically and subsequently identify their protein composition by mass spectrometry. We grew a five-litres culture of *INN1-TAP CHS2-9MYC* cells, together with *INN1 CHS2-9MYC* control cells that expressed the TAP tag under the control of the *TET* promoter. Both cultures were grown at 24°C, synchronised in G1 phase of the cell cycle by the addition of mating pheromone and cells were then released from G1 arrest for 105 minutes to focus on the time when the localisation of Inn1 and Chs2 at the cleavage site peaks. Initially, after making cell extracts, Inn1-TAP (or TAP tag in the control) were pulled down and subsequently Chs2-9MYC was immunoprecipitated from the material generated in the first step. This method facilitated the specific enrichment of Inn1-Chs2 complexes, as well as any proteins interacting with them at this point during cell division [46]. First, we confirmed by immunoblotting the presence of both Inn1 and Chs2 in our final purified material (Fig 3Ai). To identify in an unbiased fashion other factors that might regulate Chs2 activity, both purified samples were run in polyacrylamide gels and the lanes were cut into 10 bands and analysed by mass spectrometry (Fig 3Aii). A specific set of proteins that interact with Inn1-Chs2 complexes and are known core components of the budding yeast actomyosin ring was found: the sole and essential IQGAP protein Iqg1; the F-BAR domain containing protein Hof1; the type II myosin, Myo1 and Cyk3 protein, which contains a transglutaminase-like domain and an SH3 domain (Fig 3Aii). The interactions were subsequently confirmed by immunoblotting (Fig 3Aiii), using antibodies we raised against Cyk3 (S1C Fig) and Inn1 [20]. In addition, to test whether this set of proteins could be isolated immunoprecipitating another component of that newly identified complex, we pulled down protein Iqg1 fused to HA from cells going through cytokinesis synchronously, as explained above. Then, we used antibodies against Chs2 that we raised (S1D Fig), together with antibodies against Inn1 and Cyk3, to confirm that Iqg1 indeed interacted with proteins isolated in our systematic analysis (Fig 3B), in agreement with past observations of binary interactions amongst these factors [25] [19] [31] [32] [33] [34] [29] [30] [27]. These findings suggest that Inn1, Chs2, Iqg1, Hof1, Myo1 and Cyk3 interact during cytokinesis, to form complexes that coordinate actomyosin ring contraction, plasma membrane ingression and primary septum formation. We propose to name these complexes ‘ingression progression complexes’ or IPCs. To determine when during the cell cycle IPC components interact, the type II myosin Myo1 was immunoprecipitated from extracts of cells that had been arrested in G1 phase, S phase or were going through cytokinesis synchronously (Fig 3C). We found that Myo1 only interacted with IPC components at the end of the cell cycle, which is consistent with a key role of IPCs during cytokinesis (Fig 3C).

### Cyk3 binds directly to chitin synthase Chs2 and both proteins, together with Inn1, form a stable complex

**Fig 4:**
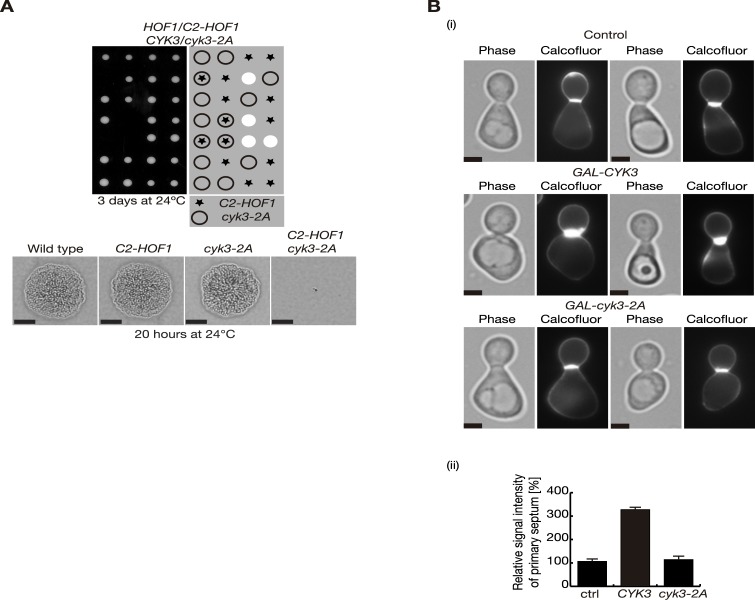
Transglutaminase-like domain of Cyk3 is important to stimulate chitin synthesis during cell division. **(A)** Tetrad analysis of the meiotic progeny from the indicated diploid strain (YMF669) shows that inactivation of the transglutaminase-like domain of Cyk3 (*cyk3-2A* allele, which contains two point mutations, D578A and H563A) is lethal when combined with *C2-HOF1*. Scale bars correspond to 20μm. **(B)** Control (YMF505), *GAL-CYK3* (YIMP235) and *GAL-cyk3-2A* (YMF576) cells were grown in YPRaff medium at 24°C, synchronised in G1 with alpha factor, and cells were released in YPGal for 135 minutes in the presence of calcofluor to visualise primary septum deposition (i). The relative signal intensity of primary septum was measured for 100 cells and compared to control cells, where signal intensity was set to 100% (ii). Scale bars correspond to 2μm.

Amongst the components of the IPCs, the Cyk3 protein is poorly characterised and might play a direct role in the regulation of Chs2 chitin synthase activity associated with Chs2, although the molecular details are unclear. Genetic studies have shown that increased doses of Cyk3 complemented defects associated with cytokinesis mutants *myo1*, *iqg1*, *inn1* and *hof1* [47] [31] [32] [42] but failed to rescue *chs2Δ* cells (S2 Fig) [42]. In addition, the overexpression of Cyk3 stimulated chitin synthesis at the division site (Fig 4B) [41] [42] and a hypermorphic allele of *CHS2* rescued defects produced by the lack of the Cyk3 protein [20]. Thus, we aimed to explore further the role of the Cyk3 subunit of IPCs in the regulation of the Chs2 chitin synthase.

Cyk3 contains two domains, an N-terminal SH3 domain and a transglutaminase-like domain located in the second half of the protein. We initially used the yeast two-hybrid assay to determine whether the Cyk3 SH3 interacted with a truncated version of Chs2 lacking the transmembrane domain (Chs2-1-629; S3 Fig). We found that the Cyk3 SH3 domain did not interact with chitin synthase Chs2, although it did interact with the C-terminus of Inn1 (S3 Fig) [31] [32] [33] [30].

The catalytic core of the active transglutaminase domains has three conserved active residues that form the catalytic triad: cysteine, histidine, and aspartic acid. The catalytic triad of the fungal Cyk3 proteins is unusual since it contains the conserved histidine and aspartic acid, but it lacks the conserved catalytic cysteine (S6A Fig) [48] [49]. To examine the role of the transglutaminase-like domain of Cyk3 in more detail, the conserved histidine and aspartic acid, which are well conserved in orthologues of Cyk3 in other eukaryotic species, were mutated to alanines (H563A and D578A; hereafter called *cyk3-2A*) (Figs 4 and S6A). To analyse the function of the Cyk3 transglutaminase-like domain in budding yeast we used cells in which the C2 domain of Inn1 was fused to the actomyosin ring component Hof1, since we have previously reported that *CYK3* becomes essential in these cells [20]. *C2-HOF1* strain grew as rapidly as a wild-type strain and did not display any detectable defects in cell division [19]. The meiotic progeny of *C2-HOF1 cyk3-2A* diploid cells was then analysed by tetrad analysis. We found that *cyk3-2A* was synthetically lethal with *C2-HOF1*, which suggested that the conserved residues H563 and D578 in the tranglutaminase-like domain are important for maintaining the function of Cyk3 (Fig 4A).

Overexpression of Cyk3 stimulated chitin synthesis at the division site (Fig 4B) [41] [42] and seemed to have no effects on cell cycle progression and Chs2 localisation (S4 Fig). To investigate whether the transglutaminase-like domain mutant *cyk3-2A* conserved the ability to increase the primary septum formation, *chs3Δ* strains overexpressing *CYK3* or *cyk3-2A*, together with control were grown at 24°C and the cells were synchronised in G1 phase. They were then released from G1 arrest into medium containing calcofluor to stain the primary septa and galactose to allow the overexpression of either Cyk3 or Cyk3-2A. Cells were collected 135 minutes after the release when the percentage of cells containing primary septa peaks. Subsequently, samples were used to examine the presence of primary septum at the division site by fluorescence microscopy. Cells overexpressing Cyk3-2A contained similar levels of primary septum as control cells (Fig 4B) unlike cells overproducing Cyk3, whose primary septa were 3 fold more intense (Fig 4B). Taken together, these observations suggest that the transglutaminase-like domain of Cyk3 is important to stimulate chitin synthesis during cell division in budding yeast.

**Fig 5:**
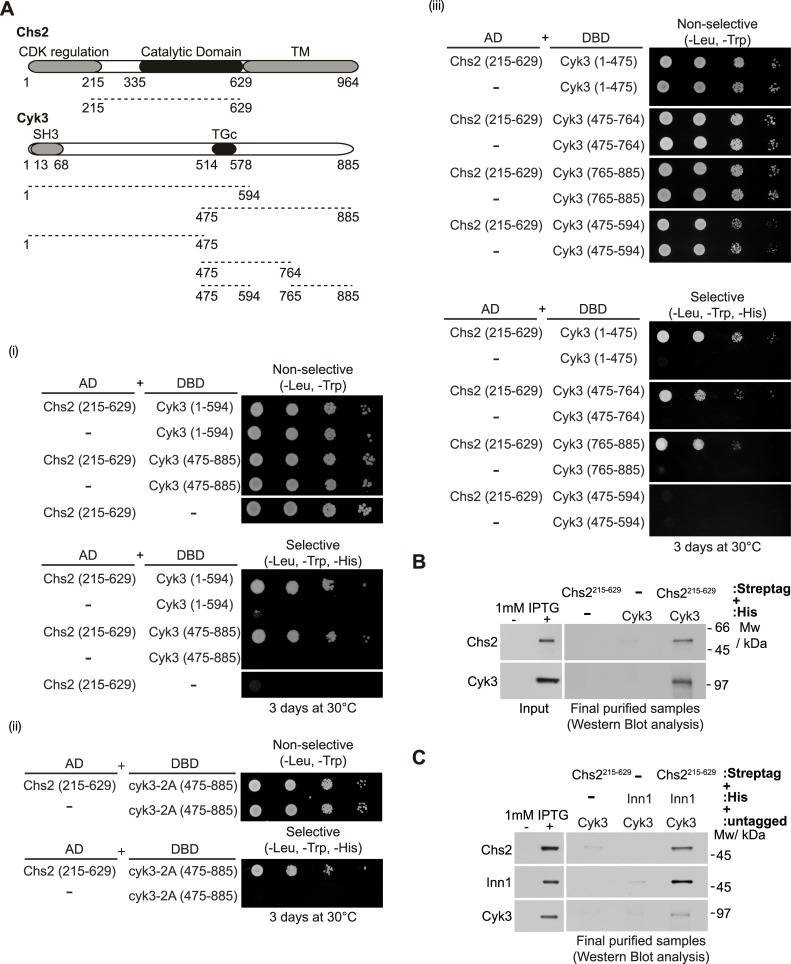
Cyk3 binds directly to Chs2 via multiple interaction sites. **(A)** Summary of yeast two-hybrid interactions between the fragment of Chs2 containing the catalytic domain (Chs2-215-629) and fragments of Cyk3. **(B)** Full-length Cyk3 interacts directly with Chs2-215-629. Pairs of *E*. *coli* cell cultures expressing 6His-tagged-Cyk3 and Strep-tag-Chs2-215-629 were mixed and used to purify putative protein complexes via Strep-Tactin Superflow and Ni-NTA agarose resins, following the scheme depicted in S1A Fig. The final purified fractions were analysed by SDS-PAGE and the tagged proteins were detected with anti-Streptag or anti-His antibodies. **(C)** Inn1, Cyk3 and the catalytic domain of Chs2 form a stable complex. Experiment was performed in a similar way to what is presented in (B) (see Materials and Methods). Cultures were mixed so that the cell extract would contain three recombinant proteins (6His-tagged-Inn1, Strep-tag-Chs2-215-629 and untagged Cyk3). In the case of controls, a culture with an empty vector was mixed with the corresponding cultures expressing the other two recombinant proteins. Chs2 and Inn1 tagged proteins were detected with anti-Streptag or anti-His antibodies respectively, and Cyk3 protein was detected with anti-Cyk3 antibodies.

To explore the possibility that Cyk3 might interact with Chs2 and therefore could be important for its chitin synthase activity, we performed a yeast two-hybrid assay with two different fragments that contained the transglutaminase-like domain of Cyk3 (Cyk3-1-594 and Cyk3-475-885) against the Chs2 truncation mentioned above that includes its catalytic domain (Chs2-215-629) (Fig 5Ai) or the fragment of Chs2 that only lacks the transmembrane domain (Chs2-1-629) (S5 Fig). We determined that both Cyk3 truncations were clearly able to interact with Chs2 (Fig 5Ai; S5 Fig). To study whether the transglutaminase-like domain mutant *cyk3-2A* conserved the ability to interact with Chs2, we carried out a yeast two-hybrid assay as above (Fig 5Aii; S5 Fig). We found that interaction was not abolished by mutations in the transglutaminase-like domain of Cyk3 (Fig 5Aii; S5 Fig). Thus, we aimed to determine whether the transglutaminase-like domain of Cyk3 interacts with Chs2, so we performed a yeast two-hybrid assay and found that a fragment of Cyk3 that contains precisely the transglutaminase-like domain (Cyk3-475-594) was unable to interact with Chs2-215-629 (Fig 5Aiii) or Chs2-1-629 (S5 Fig). Whereas a slightly bigger fragment of Cyk3 containing the transglutaminase-like domain (Cyk3-475-764), interacted with Chs2, which would indicate that the transglutaminase-like domain is not enough to bind to Chs2 (Fig 5Aiii). In addition, we observed that the two versions of Cyk3 that lack the transglutaminase-like domain (Cyk3-1-475 and Cyk3-765-885) are able to interact with Chs2, which showed that different domains within Cyk3 protein structure are responsible for the interaction between Cyk3 and Chs2 (Fig 5Aiii; S5 Fig). Interestingly we found that interactions are the same whether we performed the yeast two-hybrid assay with either Chs2-215-629 (Fig 5A) or Chs2-1-629 (S5 Fig), except for Cyk3-475-764 fragment. We showed that Cyk3-475-764 interacted with the fragment of Chs2 that lacks the N-terminal domain but not with Chs2-1-629, which would suggest that the N-terminal tail of Chs2 could play a role in regulating the interaction between Chs2 and Cyk3. Our findings would indicate that Cyk3 protein uses different domains to bind to chitin synthase Chs2.

We then examine whether artificial recruitment of the transglutaminase-like domain of Cyk3 to the actomyosin ring was sufficient to supply Cyk3 function. We have previously reported that *CYK3* becomes essential in cells in which the C2 domain of Inn1 was fused to the chitin synthase Chs2 (*C2-CHS2*) in the same way as when the C2 domain is fused to *HOF1* [20]. A diploid strain was created in which a copy of *CYK3* was inactivated (*cyk3-2A*) and one copy of *HOF1* had been modified so that the encoded protein was fused to the transglutaminase-like domain of Cyk3 (*TG-HOF1*). The meiotic progeny of the resultant strain was then analysed by tetrad analysis. We found that expression of the TG-Hof1 fusion protein rescued the lethal effects associated to *C2-CHS2 cyk3-2A* cells (S6B Fig), which shows that the fusion protein is able to bring the function of the transglutaminase-like domain to the site of division. Nevertheless, the transglutaminase-like domain is not the only essential function of Cyk3 in *C2-CHS2* cells, since artificial recruitment of the transglutaminase-like domain of Cyk3 to the actomyosin ring was insufficient to provide Cyk3 function in cells lacking the *CYK3* gene (S6C Fig).

To determine whether Cyk3 and Chs2 did indeed bind each other directly, we studied whether these factors were able to interact in *E*.*coli* extracts. We used *E*. *coli* cells to express 6His-Cyk3, in parallel with another strain that expressed Strep-tag-Chs2-215-629. After two consecutive purification steps, as described previously, we were able to observe that both proteins formed a stable complex (Fig 5B). Overall, these data suggest that Cyk3 interacts directly with chitin synthase Chs2 and regulates its enzymatic activity during cytokinesis, in which the transglutaminase-like domain of Cyk3 plays an important role.

The next step was to determine whether a protein fragment of Chs2 that contained the catalytic domain (Chs2-215-629) together with Inn1 and Cyk3 could form a stable complex in the absence of other eukaryotic proteins by using an *E*. *coli* expression system. We made parallel cultures of cells that expressed 6His-Inn1, Strep-tag-Chs2-215-629 and untagged Cyk3. We then mixed them and prepared a common cell extract containing the three proteins. After consecutive purification of the Chs2 fragment and Inn1, we were able to show that these factors formed a ternary complex with Cyk3 (Fig 5C). Therefore, our findings so far show that Chs2, Inn1 and Cyk3 proteins all bind directly to each other. These findings also indicate that both the transglutaminase-like domain of Cyk3 and the C2 domain of Inn1 regulate chitin synthase activity at the site of division.

### The Inn1 C-terminus is involved, together with Cyk3, in the regulation of chitin synthase activity associated to Chs2

**Fig 6:**
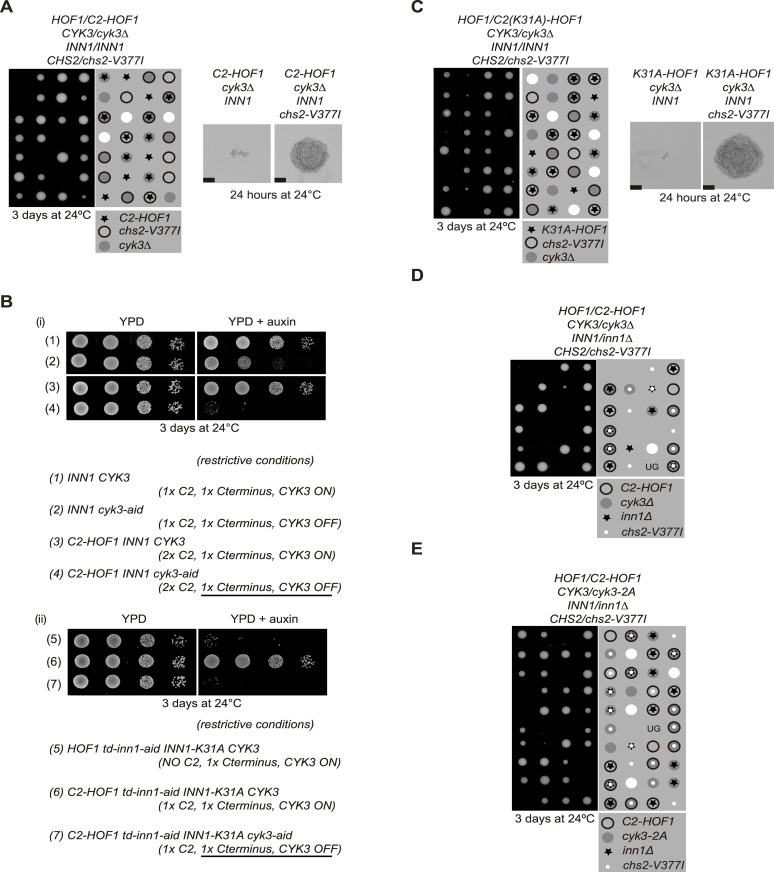
Genetic analysis reveals an important role for the C-terminus of Inn1 during cytokinesis. **(A)** Tetrad analysis of the meiotic progeny from the indicated diploid strain (YIMP11) shows that *CHS2-V377I* allows *C2-HOF1 cyk3Δ* cells to grow. Spores of the indicated genotypes were grown for 24 hours on YPD plates at 24°C. Scale bars correspond to 20μm. **(B)** Serial dilutions of strains YJW15 (1), YIMP60 (2), YIMP43 (3), YIMP41 (4), YIMP149 (5), YIMP147 (6) and YIMP142 (7) were plated on YPD medium or YPD medium containing auxin and incubated for three days at 24°C. Functional domains or proteins under restrictive conditions are indicated. **(C)** Tetrad analysis of the meiotic progeny from diploid strain (YIMP12) shows that *C2-K31A-HOF1 cyk3Δ* cells are unable to grow. Defects can be rescued by the expression of hypermorphic allele *CHS2-V377I*. Scale bars correspond to 20μm. **(D)** Tetrad analysis of the meiotic progeny from diploid strain (YIMP388) shows that the Inn1 C-terminus effect and the lack of Cyk3 can be fully bypassed by hypermorphic *CHS2* (*CHS2-V377I)*. **(E)** Tetrad analysis of the meiotic progeny from diploid strain (YIMP437) shows that C2-*HOF1 inn1Δ cyk3-2A* cells are unable to grow and defects can be rescued by the expression of hypermorphic allele *CHS2-V377I*.

We have previously found that Cyk3 is essential in cells expressing the fusion C2-Hof1 [20] despite the presence of wild-type Inn1 in these cells. We proposed that understanding why Cyk3 becomes essential in *C2-HOF1* cells could reveal the molecular details of how Inn1 and Cyk3 regulate primary septum deposition. To determine whether the problem associated with those cells was related to the function of chitin synthase Chs2, we constructed a diploid strain that lacked one copy of *CYK3* and harboured the fusion *C2-HOF1* and the hypermorphic allele of *CHS2* (Fig 6A). We found that hypermorphic Chs2 (*CHS2-V377I*) suppressed the cytokinesis defect caused by the lack of the Cyk3 protein in *C2-HOF1* cells (Fig 6A, compare double and triple mutant), which confirms that *C2-HOF1 cyk3Δ* cells fail cell division because of the defects associated with primary septum formation, despite the presence of wild-type Inn1 (Fig 6A).

One possible explanation for why Cyk3 becomes essential in *C2-HOF1* cells might be that wild-type Inn1 is unable to localise at the cleavage site, such that these cells would have C2 function (via C2-Hof1 fusion) but they would lack Inn1 C-terminus function. This hypothesis would argue that the Inn1 C-terminus and Cyk3 could share a function and cells would cope with the absence of one of them, but not with the lack of both at the same time. To determine whether wild-type Inn1 is able to localise in *C2-HOF1*, we synchronised *INN1-GFP* and *C2-HOF1 INN1-GFP* cells at 24°C in the G1 phase and then released cells to study Inn1-GFP localisation at the cleavage site. We could observe no defect in Inn1 localisation (S7A Fig).

Since *C2-HOF1* cells have two C2 domains (C2-Hof1 and full-length Inn1), second option could be that cells containing two active C2 domains might require the presence of Cyk3, presumably to regulate Chs2 function. We generated yeast strains harbouring the *C2-HOF1* fusion and the ‘auxin inducible degron’ (‘aid’) cassette on *CYK3* to conditionally inactivate Cyk3 protein [50]. We reproduced previously described synthetic lethality using tetrad analysis (Fig 6B(i); see 3 and 4). In addition, to further check this second possibility, we made strains in which cells carried a degron version of Inn1 in order to be able to deplete Inn1. In parallel these cells expressed an extra copy of *INN1*, which had been mutated to inactivate C2 function (C2-K31A), although the Inn1 C-terminus remained fully functional (*C2-HOF1 td-inn1-aid leu2*::*K31A*) (Fig 6B(ii)). After Inn1 inactivation, these cells grew as the C2 function was carried by the fusion *C2-HOF1*, despite the expression of mutated Inn1 (Inn1-K31A) (Fig 6B(ii); see 5 and 6). To determine whether Cyk3 was essential for cells expressing C2-Hof1 and Inn1-K31A, we inactivated Cyk3 and assayed cell growth after three days to show that cells died (Fig 6B(ii); see 6 and 7). Thus, we noted that the reason why *C2-HOF1 cyk3Δ* cells cannot grow is not due to the presence of extra C2 activity, but it is down to the lack of Cyk3 when cells express a functional Inn1 C-terminus (Fig 6B; see 4 and 7). Furthermore, tetrad analysis of diploid *C2-K31A-HOF1 cyk3Δ CHS2-V377I* (containing wild-type levels of Inn1) revealed that *C2-K31A-HOF1 cyk3Δ* cells are unable to form a colony (Fig 6C). Intriguingly, in those cells, despite the presence of non-functional C2 fused to *HOF1*, C2 function can be performed by wild-type copy of Inn1 (Fig 6C, see *C2-K31A-HOF1* cells). It thus appears that it is the presence of Inn1 C-terminus and the lack of Cyk3 function that are responsible for the death of *C2-K31A-HOF1 cyk3Δ* cells. This defect can be rescued by the hypermorphic allele of *CHS2* (Fig 6C, see *C2-K31A-HOF1 cyk3Δ CHS2-V377I* cells). To determine whether the lack of Inn1 C-terminus and the absence of Cyk3 can be fully bypassed by hypermorphic *CHS2*, we generated a diploid carrying the fusion *C2-HOF1*, deletions of both *CYK3* and *INN1*, together with hypermorphic allele of *CHS2* (Fig 6D). We found that the lack of Inn1 C-terminus function in *C2-HOF1 cyk3Δ inn1Δ* cells (Inn1 C2 function is carried by the fusion *C2-HOF1*) is completely rescued by increasing the chitin synthase activity associated to Chs2 (Fig 6D; the result was confirmed using a growth assay in S7B Fig). Finally, we confirmed the same observations inactivating specifically the transglutaminase-like domain of Cyk3 (*cyk3-2A*) in *C2-HOF1 inn1Δ CHS2-V377I* cells (Fig 6E). Our data would indicate that Inn1 C-terminus and Cyk3 are involved in the regulation of chitin synthase activity associated to Chs2.

**Fig 7:**
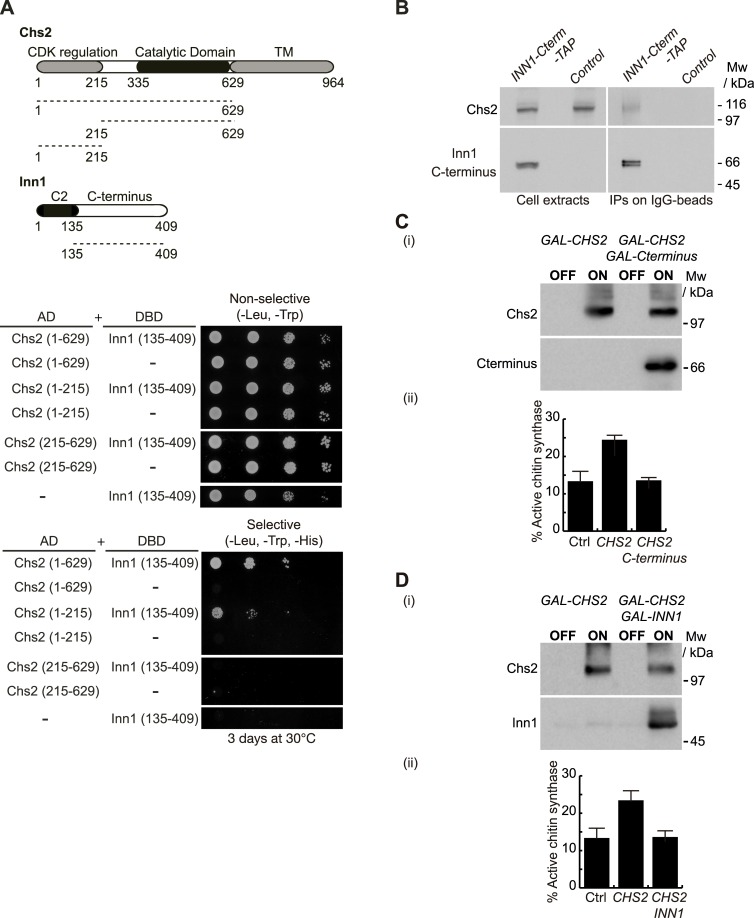
C-terminus of Inn1 interacts with Chs2. **(A)** Truncated allele of Chs2 lacking transmembrane domain (Chs2-1-629) and fragment containing the tail of Chs2 (1–215) interact in a yeast two-hybrid assay with C-terminus of Inn1 (134–409). **(B)** Control cells (YAD382) and *INN1-C-terminus-TAP* cells (YMF82) were grown at 24°C in YPD medium, arrested in G1 phase and released for 105 minutes. Inn1 C-terminus-TAP was immunoprecipitated from cell extracts on IgG beads before the detection of the indicated proteins by immunoblotting. **(C)** C-terminus of Inn1 inhibits the catalytic activity of Chs2. The protein levels of overexpressed Chs2 and C-terminus (i) and percentage of active chitin synthase (ii) in control and cells lacking Chs3 and overexpressing either *GAL-CHS2* (YMF687) or *GAL-CHS2 GAL-Cterminus* (YMF673) were determined in membranes isolated from asynchronous cultures (see Materials and Methods). **(D)** The protein levels of overexpressed Chs2 and Inn1 (i) and percentage of active chitin synthase (ii) in control and cells lacking Chs3 and overexpressing either *GAL-CHS2* (YMF687) or *GAL-CHS2 GAL-INN1* (YMF561) were determined in membranes isolated from asynchronous cultures (see Materials and Methods).

Therefore, the third possibility is that, despite the presence of C2 function (via C2-Hof1), the Inn1 C-terminus could be regulating chitin synthase Chs2 activity, in such a way that Cyk3 would be required as well. Interestingly, the C-terminus of Inn1 localises at the site of division in a manner similar to full-length Inn1 [19], which shows that the Inn1 C-terminus can still interact with components of the actomyosin ring. Using the yeast two-hybrid assay we found that the Inn1 C-terminus interacted with Chs2 (Chs2-1-629). Interestingly, the Inn1 C-terminus binds to the N-terminal tail of Chs2 (Chs2-1-215), which has been shown to be regulated by CDK activity, whereas a fragment of Chs2 that contains only its catalytic domain (Chs2-215-629) was unable to interact with the Inn1 C-terminus (Fig 7A). In order to test whether the Inn1 C-terminus could bind Chs2 *in vivo*, we generated a yeast strain in which the TAP epitope was fused to the Inn1 C-terminus. We then cultured Inn1 C-terminus-TAP and control cells (as detailed in Fig 2A) and found that Chs2 interacted with the Inn1 C-terminus (Fig 7B). To study whether the Inn1 C-terminus could regulate chitin synthase activity associated with Chs2, *chs3Δ* cells were transformed to generate strains that overexpressed Chs2 or Chs2 together with the Inn1 C-terminus in order to perform an *in vitro* chitin assay (Fig 7Ci). We found that the Inn1 C-terminus had an inhibitory effect on Chs2 activity (Fig 7Cii). We have shown that C2 overexpression had a direct positive impact on Chs2 chitin synthase activity (Fig 2C), whereas the remainder of the protein, that is the C-terminus, displayed an adverse effect on Chs2 function (Fig 7C). Subsequently, we aimed to find out how full-length Inn1 might regulate the chitin synthase activity of Chs2. Cells overexpressing Chs2, or Chs2 at the same time as full-length Inn1 (Fig 7Di), were used to assay chitin synthase activity. We found that full-length Inn1 negatively regulated Chs2 enzymatic activity (Fig 7Dii). Thus, our findings indicate that the Inn1 C-terminus blocks the ability of the C2 domain to induce chitin synthase activity associated with Chs2.

### Inn1 and Cyk3 finely regulate primary septum formation *in vivo*

**Fig 8:**
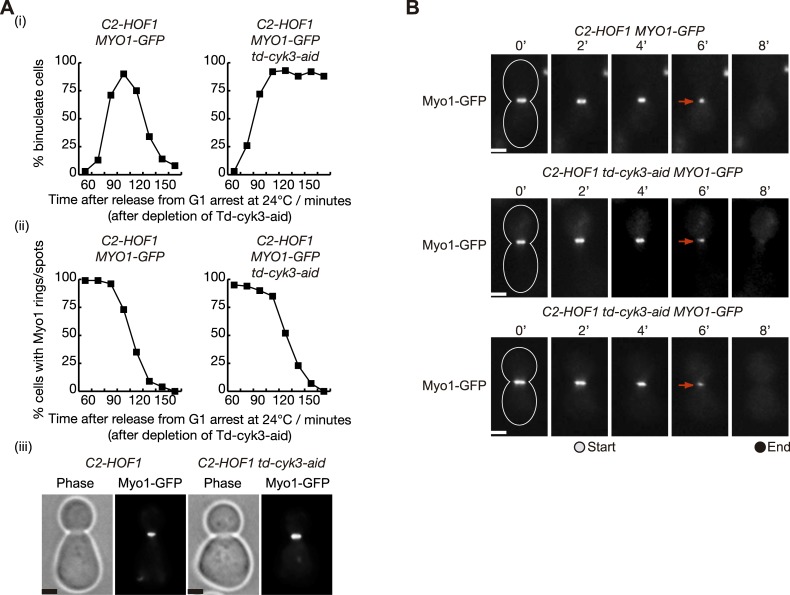
Actomyosin ring contraction is not defective in the absence of Cyk3 in *C2-HOF1* cells. **(A)** The indicated strains *MYO1-GFP C2-HOF1* (YIMP225) and *MYO1-GFP td-cyk3-aid C2-HOF1* (YIMP209) were released from G1 arrest at 24°C in YPRaff medium and cells were allowed to progress through the cell cycle at 24°C in YPGal after depleting Td-Cyk3-aid. The proportion of binucleate cells was monitored (i) in parallel with recruitment of Myo1 to the bud-neck (ii). Examples of cells with Myo1-GFP rings at the bud-neck are shown for the 105’ time-point. Scale bars indicate 2μm (iii). **(B)** The indicated strains *MYO1-GFP C2-HOF1 SPC42-EQFP* (YIMP452) and *MYO1-GFP td-cyk3-aid C2-HOF1* (YIMP209) were grown and arrested in G1 phase with mating pheromone at 24°C in YPRaff and subsequently cells were released from G1 arrest at 24°C in YPGal after depleting Td-Cyk3-aid. After cells budded and completed S-phase, nocodazole was added to synchronise the cells in G2-M-phase. Cells were then washed into fresh Synthetic Complete medium and subsequently cells were placed in the time-lapse slide to examine the localisation of Myo1 every two minutes as cells completed mitosis at 24°C (see Materials and Methods for details). Contraction of the actomyosin ring in control cells ends as a “spot”, whereas in mutant cells, the actomyosin ring disappears before that stage. Examples of cells in which time-lapse analysis was used to follow the contraction of the Myo1-GFP ring. Scale bars indicate 2μm (iii).

To determine the role of Inn1 and Cyk3 during cytokinesis, we aimed to study the defects associated with *C2-HOF1* cells in which Cyk3 was depleted. First, to investigate whether these cells had a defect in actomyosin ring formation or contraction we followed the presence of Myo1 protein at the site of division. To tightly control Cyk3 inactivation, we included a ‘heat-inducible degron’ cassette at the N-terminus of Cyk3-aid and created a double degron *td-cyk3-aid* as previously reported (S8A Fig) (‘td’ indicates the temperature sensitive degron) [51] [52] [53] [50] [20]. We grew asynchronous cultures of *C2-HOF1 td-cyk3-aid MYO1-GFP* and control cells at 24°C before synchronising cells in G1 phase with mating pheromone (Fig 8A). After the induction of both Ubr1 E3 ligase and Tir1 F-box protein, together with the addition of auxins to rapidly deplete Td-Cyk3-aid protein, cells were released at 24°C from G1 block. We observed that both mutant and control cells progressed up to anaphase in a similar manner. Unlike control cells, *C2-HOF1 td-cyk3-aid MYO1-GFP* accumulated as binucleate cells, which would reflect a failure in cell division (Fig 8Ai). Localisation of Myo1 at the site of division was observed with similar kinetics in both strains, which would indicate that mutant cells were able to assemble and contract the actomyosin ring (Fig 8Aii). To confirm the kinetics of ring contraction, time-lapse video microscopy was used (Fig 8B). *C2-HOF1 td-cyk3-aid MYO1-GFP* and control cells were grown in a similar way as for Fig 8A. After the cells had budded and completed S-phase, nocodazole was added to synchronise the cells, this time in G2-M-phase. Cells were washed into fresh medium and subsequently placed in the time-lapse slide to examine the localisation of Myo1 every two minutes as cells completed mitosis at 24°C. To ensure that both strains were treated in an identical fashion, the cultures were mixed before the cells were transferred to the time-lapse slide (the control cells expressed Spc42-eQFP and thus could be distinguished from *C2-HOF1 td-cyk3-aid* cells) (see Materials and Methods for details). Twenty-two movies each were examined for control and *C2-HOF1 td-cyk3-aid MYO1-GFP* cells, and contraction of the actomyosin ring was observed with similar kinetics (Fig 8B). The average period from the initiation of contraction to the final disappearance of the ring was similar in control and *C2-HOF1 td-cyk3-aid MYO1-GFP* cells (a mean value of 5.54 min in control cells compared with 5 min in the mutant strain). *C2-HOF1 td-cyk3-aid MYO1-GFP* cells never showed a contracted ‘spot’ as control cells. In mutant cells, the actomyosin ring disassembled before reaching the final contraction stage (‘spot’), which explains the slightly shorter contraction period in mutant cells (Fig 8B). Taken together, these experiments demonstrate that *C2-HOF1* cells in which Cyk3 has been inactivated are able to form an actomyosin ring and subsequently to contract and disassemble.

**Fig 9:**
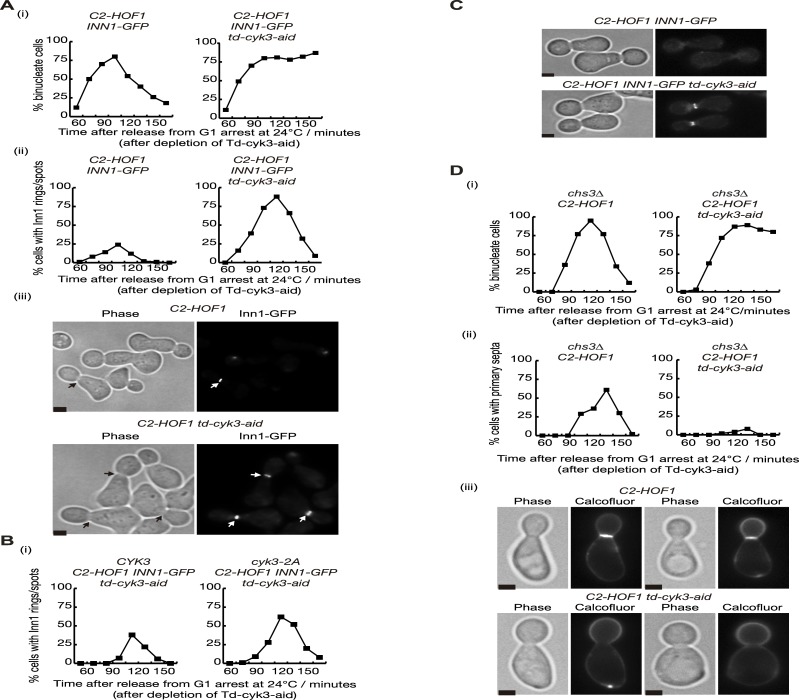
Inn1 and Cyk3 finely control primary septum deposition during cytokinesis. **(A)** The indicated strains *INN1-GFP C2-HOF1* (YIMP196) and *INN1-GFP td-cyk3-aid C2-HOF1* (YIMP198) were released from G1 arrest at 24°C in YPRaff medium and cells were allowed to progress through the cell cycle at 24°C in YPGal after depleting Td-Cyk3-aid. The proportion of binucleate cells was monitored (i) in parallel with recruitment of Inn1 to the bud-neck (ii). Examples of cells with Inn1-GFP rings at the bud-neck are shown for the 105’ time-point. Scale bars indicate 2μm (iii). **(B)** The indicated strains *INN1-GFP C2-HOF1 td-cyk3-aid CYK3* (YMF951) and *INN1-GFP C2-HOF1 td-cyk3-aid cyk3-2A* (YMF950) were grown as in (A). Recruitment of Inn1 to the bud-neck was determined. **(C)** The indicated strains from Fig 9A *INN1-GFP C2-HOF1* (YIMP196) and *INN1-GFP td-cyk3-aid C2-HOF1* (YIMP198) were released from G1 arrest at 24°C in YPRaff medium after depletion of Td-Cyk3-aid. After cells budded and completed S-phase, nocodazole was added to synchronise the cells in G2-M-phase. The recruitment of Inn1 to the bud-neck in cells arrested in G2-M phase was monitored and examples of cells with Inn1-GFP rings at the bud-neck in nocodazole-arrested cells are shown. Scale bars indicate 2μm. **(D)** Control (YIMP234) and *td-cyk3-aid C2-HOF1* (YIMP246) strains were grown as described in (A) but calcofluor was added upon release from G1 block. The proportion of binucleate cells was determined (i) and the number of cells forming primary septa stained with calcofluor was counted (ii). Examples of calcofluor-stained cells from 135 minutes after release from G1 block (iii). Scale bars correspond to 2μm.

To examine whether Inn1 localisation was altered by the lack of Cyk3 in *C2-HOF1* strain, we cultured control and *C2-HOF1 td-cyk3-aid* cells, both of which expressed Inn1-GFP. Cells were treated in the same way as for Fig 8A. We found a higher percentage of cells with Inn1-GFP accumulation in *C2-HOF1 td-cyk3-aid* cells (Fig 9A). To confirm that the lack of Cyk3 function in *C2-HOF1* cells promoted Inn1 accumulation, we transformed *C2-HOF1 td-cyk3-aid* with either *CYK3* or *cyk3-2A* alleles and cultured them in an identical way as above (Fig 9A). We showed that inactivation of the transglutaminase-like domain of Cyk3 (*cyk3-2A*) induced Inn1-GFP accumulation (Figs 9B and S8B). Since actomyosin ring contraction seemed to have no delay in *C2-HOF1 td-cyk3-aid* cells (Fig 8), which would explain the accumulation of Inn1-GFP, we aimed to determine whether Inn1-GFP localisation is slightly advanced in mutant cells. We grew control and *C2-HOF1 td-cyk3-aid* cells in the same fashion as described previously (Fig 8B). After inactivation of Td-Cyk3-aid protein, cells were synchronised in G2-M-phase with high mitotic CDK by addition of nocodazole to the culture medium. Inn1 protein is unable to be localised at the site of division before cells down-regulate CDK activity at the end of mitosis [31] [41] [54] (Fig 9C). However, we found that 33% of *C2-HOF1 td-cyk3-aid* cells were able to localise Inn1-GFP with high mitotic CDK, which indicates that inactivation of Cyk3 prompts earlier Inn1 localisation.

So far our findings indicate that Inn1 and Cyk3 formed a ternary complex with chitin synthase Chs2 (Fig 5C). In addition, our biochemical and genetic analysis show that Inn1 and Cyk3 control chitin synthase activity associated to Chs2 (Figs 6 and 7). Therefore, we aim to determine whether *C2-HOF1 td-cyk3-aid* cells have a defect in primary septum formation *in vivo*. We cultured *C2-HOF1 td-cyk3-aid* and control cells under the same conditions as indicated above for Fig 8A, but in the presence of calcofluor upon release from G1 block to stain the primary septa. We found that cells expressing C2-Hof1 together with wild-type Inn1 in the absence of Cyk3 were unable to lay down a primary septum, which would suggest that Chs2 function was impaired (Fig 9D). We were unable to determine whether Chs2 localisation occurred in *C2-HOF1 td-cyk3-aid* cells, as triple mutant cells are dead or extremely sick (*C2-HOF1 CHS2-GFP td-cyk3-aid*) (S8C Fig). However, we found that the delivery of Chs2 to the site of division seems to be similar in control and Cyk3-depleted cells, since both type of cells showed similar dynamics of the localisation of Chs2-GFP (S8D Fig) and the formation of primary septum (S8E Fig). Therefore, our data indicate that the lack of Cyk3 does not prevent Chs2 localisation and primary septum deposition.

Overall these data show that Inn1 regulates Chs2 activity at the site of division, where the C2 domain induces Chs2 function, whereas the C-terminus of Inn1 seems to have an inhibitory effect on Chs2. Our results indicate that Cyk3 counteracts this inhibitory role since Cyk3 becomes essential under conditions in which the Inn1 C-terminus plays a more relevant role, such as in *C2-HOF1* cells. In addition, Cyk3 seems not to have a role in the delivery of Chs2 vesicles to the cleavage site.

### Chs2 localisation at the division site requires the presence of IPC components

**Fig 10:**
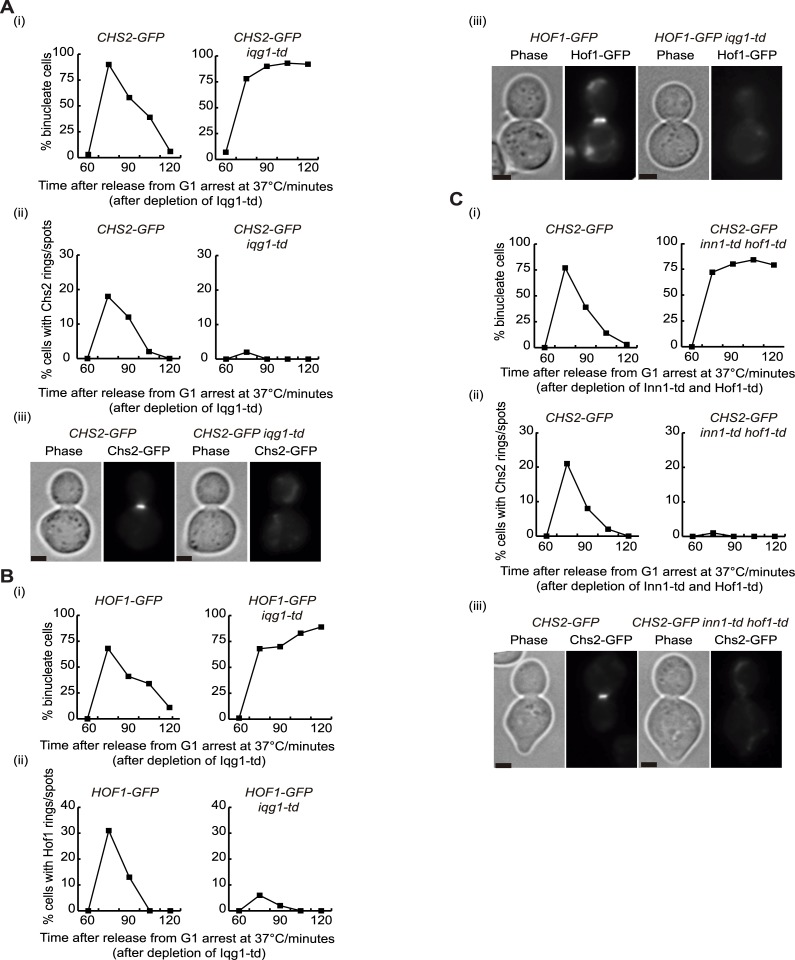
Chs2 localisation at the division site requires the presence of IPC components. **(A)** The indicated strains *CHS2-GFP* (YMF330) and *CHS2-GFP iqg1-td* (YMF305) were arrested in G1 phase at 24°C in YPRaff and then shifted to YPGal at 37°C to deplete Iqg1-td. Subsequently, cells were released to allow progression through the cell cycle. Samples were taken at the indicated times to determine the proportion of binucleate cells (i) and the percentage of cells with rings or spots of Chs2 at the cleavage site (ii). Examples of cells with Chs2-GFP rings at the bud-neck are shown for the 75’ time-point (iii). **(B)** The indicated strains *HOF1-GFP* (YASD550) and *HOF1-GFP iqg1-td* (YASD556) were grown as in (A). The proportion of binucleate cells (i) and the recruitment of Hof1 to the site of division (ii) were monitored. Examples of cells with Hof1-GFP rings at the bud-neck are shown for the 75’ time-point (iii). **(C)** The indicated strains *CHS2-GFP* (YMF330) and *CHS2-GFP inn1-td hof1-td* (YIMP230) were grown as in (A). The proportion of binucleate cells was studied (i) together with Chs2 localisation at the cleavage site (ii). Examples of cells with Chs2-GFP rings at the bud-neck are shown for the 75’ time-point (iii). Scale bars correspond to 2μm.

Chs2 protein interacts with actomyosin ring components to build the IPCs at the end of mitosis. To understand the importance of these interactions for Chs2 localisation and maintenance at the site of division we studied the fluorescence signal associated with Chs2-GFP in controls cells and in cells in which a particular actomyosin ring component had been previously inactivated. Cultures of *CHS2-GFP* and *iqg1-td CHS2-GFP* cells were grown at 24°C and cells were synchronised in G1 phase of the cell cycle with mating pheromone, before rapidly inactivating Iqg1 at 37°C. Upon release from G1 arrest at 37°C, *iqg1-td* cells completed mitosis but were unable to divide unlike the control cells (Fig 10Ai). Importantly, medial rings or contracted dots of Chs2 were not observed in the absence of Iqg1 (Fig 10Aii and 10Aiii). This shows that Iqg1 is essential for the localisation of Chs2. Additionally, it has been described that Iqg1 interacts with Hof1 and Inn1, and we have previously reported that Inn1-Iqg1 interaction is required for the Inn1 protein to be localised at the division site [19]. Our next step was therefore to determine whether Iqg1 is important for Hof1 to interact with the actomyosin ring. We grew *HOF1-GFP* and *iqg1-td HOF1-GFP* cells as detailed in Fig 10A and we found that the absence of Iqg1 caused a defect in Hof1 localisation (Fig 10B). Taken together, these experiments indicate that the Iqg1 protein is crucial for building functional IPCs at the end of mitosis in budding yeast.

To determine whether Chs2 localisation requires the presence of Hof1 or Inn1 at the site of division, we grew *CHS2-GFP* and *hof1-td CHS2-GFP* cells asynchronously at 24°C and cultured them in the same manner as described for Fig 10A. After rapid depletion of Hof1 in cells we followed Chs2-GFP localisation and found that Chs2-associated signal at the cleavage site was compromised when Hof1 protein was inactivated (S9A Fig). Subsequently, we investigated whether Chs2 localisation depends on Inn1 protein. We observed that Chs2 protein was still able to localise, although Chs2 dynamics at the site of division seemed to be affected, since we detected less Chs2 at the division site (S9B Fig). Finally, we investigated whether the lack of Hof1 and Inn1 at the same time would affect Chs2 recruitment. *CHS2-GFP* and *hof1-td inn1-td CHS2-GFP* cells were grown and, after rapid inactivation of Hof1 and Inn1, we found that Chs2 localisation at the cleavage site was entirely dependent on the presence of both Hof1 and Inn1 (Fig 10C), which is the same result as showed above for *iqg1-td* cells. Our findings would suggest that the dynamics of the chitin synthase Chs2 at the site of division in budding yeast requires the interaction with either Iqg1 protein or Hof1 and Inn1 together.

## Discussion

Our data indicate that budding yeast cells assemble at the end of mitosis protein complexes that we have named ingression progression complexes (IPCs) to coordinate actomyosin ring contraction, plasma membrane ingression and primary septum formation. The IPCs include Myo1, Iqg1, Hof1, Inn1, Cyk3 and Chs2. We propose that the IPCs indeed form the central machinery with which cells are able to coordinate cytokinetic events, which provides a mechanistic explanation for the tight coordination between them [55] [17] [8].

**Fig 11:**
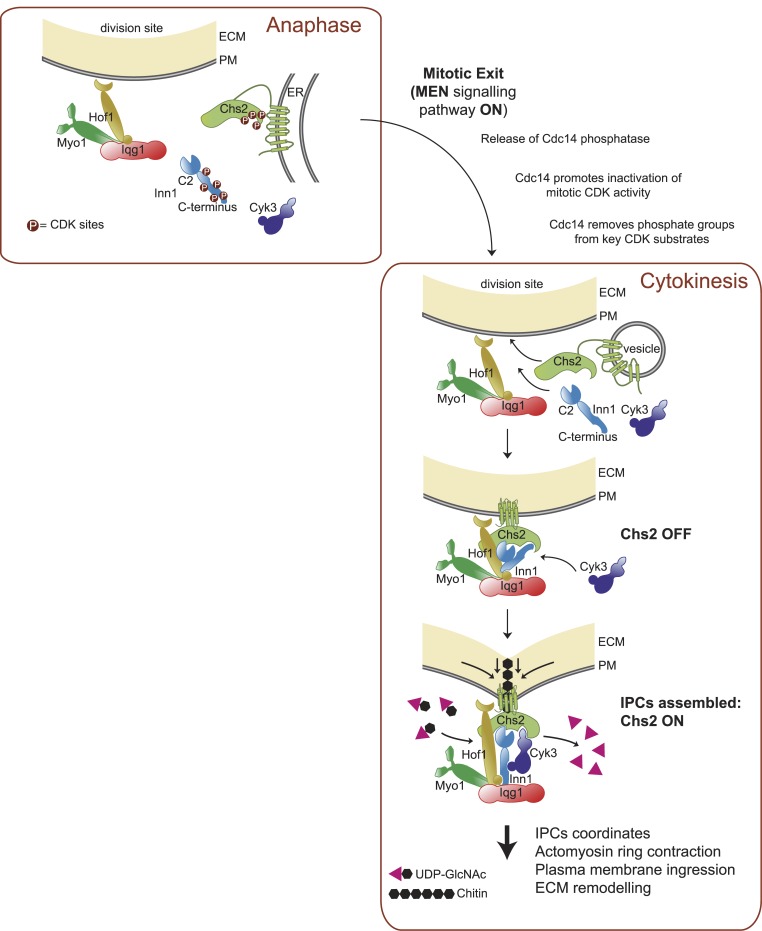
Assembly and function of IPC components during cell division in budding yeast. IPCs components are recruited to the site of division in a sequential and highly regulated manner. The type II myosin Myo1, the IQGAP protein Iqg1 and the F-BAR protein Hof1 are localised at the cleavage site before cells release the special phosphatase Cdc14 to downregulate the kinase activity associated with CDK/Cyclin complexes and to allow exit from mitosis and cytokinesis. In addition, Cdc14 removes phosphate groups from key CDK substrates such as Chs2 and Inn1, which forces both Chs2 and Inn1 to localise at the bud neck. Hof1 facilitates Chs2 incorporation to the plasma membrane at the site of division and Inn1 collaborates to maintain Chs2 at the buck-neck. Chitin synthase activity associated with Chs2 remains inactive due to the action of the C-terminus domain of Inn1. Subsequently, the C-terminus of Inn1 allows interaction with Cyk3, which counteracts C-terminus inhibition and induces, together with the C2 domain of Inn1, Chs2 enzymatic activity to lay down the primary septum. We suggest that IPCs would coordinate actomyosin ring contraction, membrane ingression and primary septum deposition for the successful completion of cytokinesis.

Our data support a model whereby IPCs are assembled in a sequential and highly regulated fashion to control first the localisation and then the activation of the chitin synthase Chs2 at the end of mitosis, which plays a key role in the tight coordination of actomyosin ring contraction, plasma membrane ingression and primary septum formation (Fig 11) [8] [37] [38] [39] [14]. Myo1 and Iqg1 serve as initial building blocks with which the other IPC components Hof1, Cyk3, Inn1 and Chs2 then interact [19] [31] [32] [33] [34] [30] [29] [27]. Specific inactivation of Myo1 or Iqg1 prevents the localisation of the other components of IPCs, which highlights their scaffolding role [19]. Moreover, IPC members commonly displayed Myo1-dependent immobility during cytokinesis, supporting further that Myo1 plays a scaffolding role in the assembly of IPCs [24].

Our findings indicate that Hof1 would facilitate Chs2 localisation at the site of division whereas Inn1 and Cyk3 are essential for the activation of chitin synthase activity. In addition to its role in promoting the formation of the primary septum, Hof1 plays an important role in the assembly of the actomyosin ring in *S*. *cerevisiae*, together with the protein Rvs167 [56] [55] [28] [29] [30]. It appears that the basic principles of action of budding yeast Hof1 and its fission yeast orthologue Cdc15 are likely to be similar, with both proteins contributing to assembly of the actomyosin ring as well as to the stability of the contracting ring and/or septum formation [57] [58] [59] [29] [30] [11]. In budding yeast it has been described that Hof1 interacts directly with Chs2 and stabilises the chitin synthase at the cleavage site [29]. Accordingly, we found that Chs2 localisation at the bud neck is clearly compromised in Hof1-depleted cells. In addition, we observed that increased expression of Chs2 rescues defects associated with the lack of Hof1 (S9C Fig). However, a hypermorphic version of Chs2 was unable to supress the cell division defect produced by Hof1 inactivation (S9D Fig), unlike what occurs with Inn1 or Cyk3-depleted cells [20]. Our findings indicate that Hof1 assists in the incorporation of Chs2 at the cleavage site and not in its activation. Our data is consistent with recent reports that showed how fission yeast cells lacking Cdc15 fail to accumulate at the cleavage site the protein that plays an analogous role to Chs2 [11], namely the transmembrane glycosyltransferase Bgs1 (beta(1,3)-glucan synthase), which lays down the primary septum during cytokinesis in fission yeast cells [9] [60].

Chs2 appears to be delivered to the plasma membrane in an inactive form and is then activated *in situ* by a mechanism that has not previously been understood [31] [19] [20] [32] [40]. Our data indicate that the Inn1 and Cyk3 proteins are indeed directly responsible for such activation (Fig 11). The interaction of Chs2, Inn1 and Cyk3 would require the inactivation of mitotic forms of Cyclin Dependent Kinase (CDK) and the dephosphorylation of CDK targets such as Chs2 and Inn1 by the Cdc14 phosphatase [38] [39] [33] [54] [61] [62].

Our model proposes that Inn1 binds to Chs2 at the end of mitosis, but the C-terminus of Inn1 keeps Chs2 chitin synthase inactive. It has been reported that a version of Chs2 that lacks its N-terminal domain show high levels of chitin synthase activity (Martinez-Rucobo et al 2009), which suggests that the N-terminal tail of Chs2 (Chs2-1-215) negatively regulates its own activity. Interestingly, our data indicate that the C-terminus of Inn1 binds precisely to the N-terminal tail of Chs2 (Fig 7A) and, consistently with these data, we described how the C-terminus of Inn1 blocks chitin activity associated to Chs2. In addition, it seems that the N-terminal tail of Chs2 could be regulating as well the interaction between Chs2 and Cyk3 (Fig 5A). Cyk3 becomes incorporated to the IPCs and precisely releases chitin synthase activity of Chs2 from such a block and consequently the C2 domain of Inn1 acts in conjunction with Cyk3 to activate the catalytic domain of Chs2 (Fig 11). In addition to its catalytic activity, Chs2 has attractive features to serve as an anchor between the actomyosin ring and the plasma membrane, since it is the only component of the IPCs that has a transmembrane domain embedded in the plasma membrane. It appears that, in budding yeast, having such a physical link with the plasma membrane is not sufficient for ingression, since cells need active glycosyltransferase for extracellular matrix remodelling and its coordination with actomyosin ring contraction during cytokinesis [19] [20].

Interestingly, each component of budding yeast IPCs has an orthologue in fission yeast cells with a role during cytokinesis, although the molecular mechanism by which they regulate cell division is not yet understood in all cases [60] [22] [63] [58] [49]. Fission yeast cells have orthologues of Inn1 and Cyk3, namely Fic1 and Cyk3, which share the same structure as their budding yeast counterparts and, in addition, they have been described to play a role during cytokinesis [58] [49]. The Fic1 protein interacts with Cdc15 and adds structural integrity to the actomyosin ring and prevents it from collapsing during cell division [58], whereas fission yeast Cyk3 has been suggested to play a role in coupling actomyosin ring contraction and primary septum formation, although the molecular mechanism remains unclear [49]. The presence of chitin in *S*.*pombe* septum is uncertain, but instead it has been proposed that *S*.*pombe* Chs2 would play a structural role and would be required for proper actomyosin ring contraction and stability [64]. Intriguingly, in fission yeast the glycosyltransferase Bgs1 could play the same role as Chs2 in budding yeast, although the polysaccharide that Bgs1 produces is different (Chs2 synthesises chitin, which is a polymer of N-acetylglucosamine; Bgs1 synthesises glucan, which is a polymer of D-Glucose). Bgs1 is an integral membrane protein with its catalytic domain located at the cytoplasmic side of the membrane like Chs2 [9] [60]. Interestingly, the lack of Bgs1 promotes actomyosin ring sliding along the plasma membrane, which supports the idea that Bgs1 could function as an anchor between the actomyosin ring and the plasma membrane [11] [65].

How human cells perform the coordination of actomysin ring contraction, plasma membrane ingression and ECM remodelling remains largely unknown. Taking into account the conservation of the basic cytokinetic mechanisms [22] [60] [3], it will be interesting to determine whether glycosyltransferases also play a role during cytokinesis in higher eukaryotes.

## Materials and Methods

### Construction and growth of yeast strains

The strains used in this study are listed in S1 Table. Yeast cells were grown in rich medium (1% Yeast Extract, 2% peptone, 0.1 mg per ml adenine) supplemented with 2% glucose (YPD), 2% Raffinose (YPRaff) or 2% Galactose (YPGal) as the carbon source with the exception of cells for time-lapse video microscopy, for which we used Synthetic Complete medium at the end of the experiment. We arrested cells in the G1 phase of the cell cycle by the addition of alpha factor mating pheromone to the medium at a final concentration of 7.5 μg per ml. We arrested cells in the G2-M phase of the cell cycle by the addition of nocodazole to the medium at a final concentration of 5 μg per ml. To degrade proteins fused to the ‘heat-inducible degron’ and ‘auxin-inducible degron’ we followed procedure described previously [66] [50]. In experiments with temperature sensitive degron strains and for strains expressing fused proteins (*C2-HOF1*, *C2-CHS2 and TG-HOF1)*, 0.1mM CuSO_4_ was included in the growth medium, as all of them were expressed from the *CUP1* promoter. To stain primary septa of living cells, calcofluor was added when specified 30 minutes after release from G1 block to a final concentration of 0.05 mg per ml and culture was incubated further for at least 60 minutes.

### Two-hybrid analysis

Two-hybrid analysis was performed using the vectors pGADT7 and pGBKT7 (Clontech). Cells were grown for two or three days at 30°C on Synthetic Complete medium lacking leucine and tryptophan (non-selective) or lacking leucine, tryptophan and histidine (selective).

### Expression and purification of recombinant proteins in *E*.*coli*

The plasmids used in this study to express recombinant proteins in *E*.*coli* are based on the ‘pET’ series (Novagen) and are listed in S2 Table. To isolate recombinant protein complexes from extracts of *E*.*coli* cells, we followed the scheme illustrated in S1A Fig and as it was described previously [30]. The various protein fragments were expressed individually as ‘Streptag’ or ‘6His-tag’ fusions. Cells containing each of the fusions were grown at 37°C, after which the expression of the recombinant protein fragments was induced with 1 mM IPTG. Subsequently, the cultures were mixed, so that each cell extract would contain two recombinant proteins (Figs 1B, 1C, 1D, 2A(ii), 2A(iii) and 5B) or three recombinant proteins (Fig 5C). In the case of the controls, a culture with an empty vector was mixed with the corresponding cultures expressing recombinant proteins. The Streptag-fusions were then isolated from the cell extracts in 1 ml of Strep-Tactin Superflow (2-1206- 025, IBA GmbH), before elution with 2.5 mM d-Desthiobiotin (D1411, Sigma). The eluted material was then diluted and incubated with 1 ml of Ni-NTA Agarose (30230, Qiagen), and bound protein complexes were eluted with sequential 0.5 ml aliquots of buffer containing 250 mM imidazole. Following the addition of 3X Laemmli buffer to the eluted samples, 20 μl of each purified sample was resolved by SDS-PAGE.

### Immunoprecipitation of protein complexes from yeast cell extracts

To monitor the association of proteins in yeast cell extracts we followed methods previously described [67] [46] with slight modification as cell extracts were spun down at 20 000 x g. We have isolated tagged proteins by immunoprecipitation with magnetic Dynabeads M-270 Epoxy (Invitrogen) coupled to rabbit anti-sheep IgG (Sigma S-1265), 9E10 anti-MYC monoclonal antibody (Cancer Research Technology) or 12CA5 anti-HA monoclonal antibody (Cancer Research Technology).

### Detection of proteins

We detected the indicated proteins by immunoblotting with previously described polyclonal antibodies to Inn1 [20] or by using polyclonal anti-FLAG antibody (Sigma F-7425), or monoclonal 9E10 (anti-MYC), or 12CA5 (anti-HA). To detect Chs2 (rabbit polyclonal) and Cyk3 (sheep polyclonal), we raised polyclonal antibodies to 25 kDa portions of each protein (S1C and S1D Fig), expressed as His-tagged recombinant proteins in *E*.*coli* and purified in a denatured form. The TAP tag was detected using the rabbit peroxidase anti-peroxidase complex (Sigma P-2026). For mass spectrometry analysis of protein content, the digested peptides were analysed by nano LC/MS/MS with an ‘Orbitrap Velos’ (ThermoFisher) and the data were processed as described previously [68] [69].

### Measuring the chitin synthase activity associated with Chs2

Cell membrane isolation and chitin synthase activity assays were performed as described previously [20], clearly detecting chitin synthase activity associated to Chs2 (S10A Fig) In isolated cell membranes Chs2 is nearly inactive, unless protease treatment is used to bypass inhibition [14]. To clearly identify whether full-length Inn1, Inn1 C2 or Inn1 C-terminus had an effect on chitin synthase associated with Chs2, we plotted the percentage of active chitin synthase calculated as percentage of chitin synthase activity (without trypsin) compared to the maximum chitin activity reached by the same sample (with trypsin): (chitin synthase activity without trypsin / chitin synthase activity with trypsin) x100. Error bars represent SEM values calculated for each of the experiments.

### Flow cytometry and binucleate cell analysis

Samples used to measure the DNA content were fixed with 70% ethanol. Subsequently samples were processed and stained with propidium iodide after RNA digestion, as described previously [53]. The proportion of binucleate cells was determined by observing the same samples under the microscope as those employed for flow cytometry [70]. We examined 100 cells for each sample.

### Microscopy

Pictures of cells and colonies on agar plates were taken after 24 hours (YPD medium) or 30 hours (YPGal medium) with a Nikon CoolPix 995 camera attached to a Nikon Eclipse E400 microscope. We used calcofluor (Fluorescent Brightener 28; Sigma; F3543-1G) to stain the primary septa of live cells. We tested that there was a direct correlation between calcofluor staining and chitin synthase activity associated to Chs2 *in vivo* (S10B Fig). Calcofluor was added 30 minutes after release from G1 block to a final concentration of 0.05 mg per ml and the culture was further incubated for at least 60 minutes. Calcofluor-stained cells were observed live. To quantify primary septum deposition, we examined 100 cells with primary septum for each sample, and measure the relative signal intensity of the primary septum using Image J software [71]. To observe GFP-tagged proteins, the cells were fixed with 8% formaldehyde for 10 minutes and subsequently washed twice with PBS [19]. We examined 100 cells for each sample. Phase contrast and fluorescence microscopy images of cells grown in liquid culture were obtained with a Nikon A1R Microscope and an Orca R2 camera (Hamamatsu) with objective lens Plan Apo TIRF 100x oil DIC 1.49NA, and LightLine single-band filter set FITC Semrock. The illumination source was the Nikon Intensilight C-HGFIE (ultrahigh Presure 130W Mercury lamp), and we used NIS elements software. We analysed eleven z-sections with a spacing of 0.375 μm to facilitate the examination of the whole cell for all experiments. In all cases, the exposure time, sensor gain, and digital adjustments were the same for the control and experimental samples.

Time-lapse video microscopy was performed using DeltaVision system with Olympus IX-71 microscope and CoolSNAP HQ2 Monochrome camera. The objective lens was Plapon 60X0 1.42 NA. The illumination source was the 300W xenon system with liquid light guide, and we used Softworx Resolve 3D acquisition software. Cells were grew in an IBIDI cells in focus 15 micro-slide (8 well 80827 glass bottom). The base of the time-lapse chamber is formed by a glass coverslip that we coated with a 5 mg per ml solution of the lectin Concanavalin A (Sigma L7647), and then washed with water and dried for 30 minutes. We analysed 10 z-sections with a spacing of 0.4 μm.

The microscopy data were deconvolved, except for cells stained with calcofluor, using Huygens (SVI) according to the “Quick Maximum Likelihood Estimation” method and a measured point spread function. The deconvolved data set was viewed with Image J software [71].

## Supporting Information

S1 Fig **(A) Expression and purification of recombinant proteins in E. coli.** Scheme explaining how the indicated protein fragments were expressed in cultures of *E*. *coli* cells, and then mixed to allow the purification of protein complexes. After induction with IPTG, pairs of cultures were mixed as indicated below, and used to purify protein complexes between the induced proteins, via Strep-Tactin Superflow and Ni-NTA agarose resins (see Materials and Methods). **(B)** Overexpression of the C2 domain of Inn1 does not change the localisation of Chs2. Control *CHS2-GFP* (YASD819) and *GAL-C2 CHS2-GFP* (YMF660) cells were grown in YPRaff medium at 24°C and synchronised in G1 with alpha factor. Subsequently, cells were released in YPGal from G1 block and proportion of binucleate cells (i) and the recruitment of Chs2 to the site of division (ii) were monitored. **(C)** Specificity of Cyk3 antibodies. An affinity-purified sheep polyclonal antibody specific for Cyk3 was tested by immunoblotting using protein extracts from the wild-type and *CYK3-GFP* (YASD641) strains. **(D)** Specificity of Chs2 antibodies. An affinity-purified rabbit polyclonal antibody specific for Chs2 was tested by immunoblotting using protein extracts from the wild-type and *CHS2-GFP* (YASD819) strains.
(EPS)

S2 Fig Overproduction of Cyk3 does not rescue the lack of Chs2. **(A)** Tetrad analysis of the meiotic progeny from the indicated diploid strain (YIMP255) shows that *GAL-CYK3* does not allow *chs2Δ* cells to grow. Spores of the indicated genotypes were grown for 30 hours on YPGal plates at 24°C. Scale bars indicate 20μm. **(B)** Serial dilutions of the control (YIMP267), *chs2-aid* (YAD394) and *chs2-aid GAL-CYK3* (YIMP265) strains were plated on YPGal medium or YPGal medium containing auxin and incubated for four days at 24°C.
(EPS)

S3 Fig SH3 domain of Cyk3 is unable to interact with Chs2. Summary of yeast two-hybrid data between Chs2 and Cyk3. The Inn1 C-terminus fragment was used as a control to show the interaction with the Cyk3 SH3 domain.
(EPS)

S4 Fig Overexpression of Cyk3 or Cyk3-2A does not have an effect on cell cycle progression and Chs2 localisation. *CHS2-GFP GAL-CYK3* (YMF610) and *CHS2-GFP GAL-CYK3* (YIMP423) cells, were grown in YPRaff medium at 24°C and synchronised in G1 phase with mating pheromone. Cells were released from G1 arrest at 24°C on YPGal medium to allow them to progress through the cell cycle. The proportion of binucleate cells was monitored (i) in parallel with the recruitment of Chs2 to the bud-neck (ii). Examples of cells with Chs2-GFP rings at the bud-neck are shown for the 105’ time-point (iii). Scale bars correspond to 2μm. For each timepoint, 100 cells were examined to determine the percentage of Chs2-GFP localisation.
(EPS)

S5 Fig Chs2 interacts with Cyk3. Summary of yeast two-hybrid interactions between the fragment of Chs2 lacking only transmembrane domain (Chs2-1-629) and fragments of Cyk3.
(EPS)

S6 Fig Fusion of transglutaminase domain to *HOF1* is enough to partially rescue defects associated with *cyk3-2A C2-CHS2* cells but not to rescue *cyk3Δ C2-CHS2* cells. **(A)** Orthologues of the budding yeast Cyk3 in the indicated fungal species were identified by PSI-BLAST searches, aligned with ClustalW software (http://seqtool.sdsc.edu/CGI/BW.cgi) and displayed using ‘Boxshade’. The figure shows their transglutaminase-like domain and the conserved residues. All the proteins share conserved histidine and aspartic acid as in the transglutaminase catalytic triad, which is the hallmark of the family of transglutaminase enzyme. However they lack the cysteine residue present in the catalytic triad. **(B)** Tetrad analysis of the meiotic progeny from the indicated diploid strain (YMF960) shows that *TG-HOF1* allows *cyk3-2A C2-CHS2* cells to grow. Spores of the indicated genotypes were grown for 24 hours on YPD plates at 24°C. Scale bars correspond to 20μm. **(C)** Tetrad analysis of the meiotic progeny from the indicated diploid strain (YMF953) shows that *TG-HOF1* does not rescue defects associated with *cyk3Δ C2-CHS2*. Spores of the indicated genotypes were grown for 24 hours on YPD plates at 24°C. Scale bars correspond to 20μm. “UG” denotes ungerminated spore.
(EPS)

S7 Fig C-terminus of Inn1 plays a role during cytokinesis. **(A)** The indicated strains *INN1-GFP* (YMF373) and *C2-HOF1 INN1-GFP* (YIMP196) were released from G1 arrest at 24°C in YPD medium and allowed to progress through the cell cycle. The proportion of binucleate cells was monitored (i) in parallel with the recruitment of Inn1 to the bud-neck (ii). **(B)** Serial dilutions of strains YIMP334 (1), YIMP41 (2), YIMP329 (3), YIMP324 (4), YIMP242 (5), YIMP240 (6) and YIMP310 (7) were plated on YPD medium or YPD medium containing auxin and incubated for two days at 24°C.
(EPS)

S8 Fig Lack of Cyk3 function induces accumulation of Inn1 at the bud neck. **(A)** Cultures of control cells (YMF334) and *C2-HOF1 td-cyk3-aid GAL-UBR1 GAL-OsTIR1* (YMF356) were grown at 24°C in YPRaff medium before being shifted to YPGal medium containing auxin for the indicated times. The DNA content was monitored throughout the experiment by flow cytometry, and images of cells were captured nine hours after depleting Td-Cyk3-aid. Scale bars indicate 5μm. **(B)** The indicated strains *INN1-GFP C2-HOF1 td-cyk3-aid CYK3* (YMF951) and *INN1-GFP C2-HOF1 td-cyk3-aid cyk3-2A* (YMF950) were released from G1 arrest at 24°C in YPRaff medium and cells were allowed to progress through the cell cycle at 24°C in YPGal after depleting Td-Cyk3-aid. Examples of cells with Inn1-GFP rings at the bud-neck are shown for the 105’ time-point. Scale bars indicate 2μm. **(C)** Tetrad analysis of the meiotic progeny from the indicated diploid strain (YIMP428) shows that cells harbouring *C2-HOF1 td-cyk3-aid CHS2-GFP* are dead or extremely sick. **(D)** Control (YIMP206) and *td-cyk3-aid CHS2-GFP* (YMF343) strains were released from G1 arrest at 24°C in YPRaff medium and cells were allowed to progress through the cell cycle at 24°C in YPGal after depleting Td-Cyk3-aid. The proportion of binucleate cells was determined (i) and the localisation of Chs2-GFP was monitored (ii). **(E)** Control (YIMP248) and *td-cyk3-aid* (YIMP247) strains were released from G1 arrest at 24°C in YPRaff medium and cells were allowed to progress through the cell cycle at 24°C in YPGal after depleting Td-Cyk3-aid in the presence of calcofluor. The proportion of binucleate cells was determined (i) and the number of cells forming primary septa stained with calcofluor was counted (ii).
(EPS)

S9 Fig Hof1 and Inn1 are important for Chs2 to be detected at the division site during cytokinesis. **(A)** The indicated strains, *CHS2-GFP* (YMF330) and *CHS2-GFP hof1-td* (YIMP189), were arrested in G1 at 24°C then shifted at 37°C and allowed to progress through the cell cycle after Hof1-td had been depleted. The proportion of binucleate cells was monitored (i) together with recruitment of Chs2 to the bud-neck (ii). **(B)** The indicated strains, *CHS2-GFP* (YMF330) and *CHS2-GFP inn1-td* (YASD689) were grown as in (A). The proportion of binucleate cells (i) and the localisation of Chs2 to the bud-neck (ii) were determined. **(C)** Serial dilutions of control (YIMP273), *hof1-td* (YASD681) and *hof1-td GAL-CHS2* (YIMP272) cells were plated on YPGal medium and incubated for four days at 24°C or for three days at 37°C to deplete Hof1-td protein. **(D)** Serial dilutions of control (YIMP254), *hof1-td* (YASD681) and *hof1-td CHS2-V377I* (YIMP253) cells were plated on YPD medium or YPGal medium and incubated for three days at 24°C or 37°C to deplete Hof1-td protein.
(EPS)

S10 Fig Chs2 is the effector of the chitin synthase activity in cells. **(A)** The amount of chitin synthase activity was measured without protease treatment using membranes isolated from asynchronous cultures in control (YMF891), *chs2-aid CHS2-V377I* (YMF893) and *chs2-aid CHS2-V377I-D562A* (YMF894). **(B)** Control (YMF505) and *CHS2-V377I* (YMF694) cells were grown in YPRaff medium at 24°C and synchronised in G1 with alpha factor. Subsequently, cells were released in YPGal for 135 minutes from G1 block in the presence of calcofluor to visualise primary septum deposition. Examples of these cells are shown in (i). Scale bars correspond to 2μm. The relative signal intensity of primary septum was measured for 100 cells and compared to control cells, where signal intensity was set to 100% (ii).
(EPS)

S1 Table Strains used in this study (all based on W303). (DOC)

S2 Table Plasmids used to express recombinant proteins in *E*.*coli*. (DOC)
