# Correction: Ingression Progression Complexes Control Extracellular Matrix Remodelling during Cytokinesis in Budding Yeast

**DOI:** 10.1371/journal.pgen.1005988

**Published:** 2016-04-07

**Authors:** The PLOS Genetics Staff

Figs 2, 4 and 9 are incorrectly stretched, causing the cell sizes to be out of proportion. Please view the correctly sized figures here. The publisher apologizes for the error.

**Fig 2:**
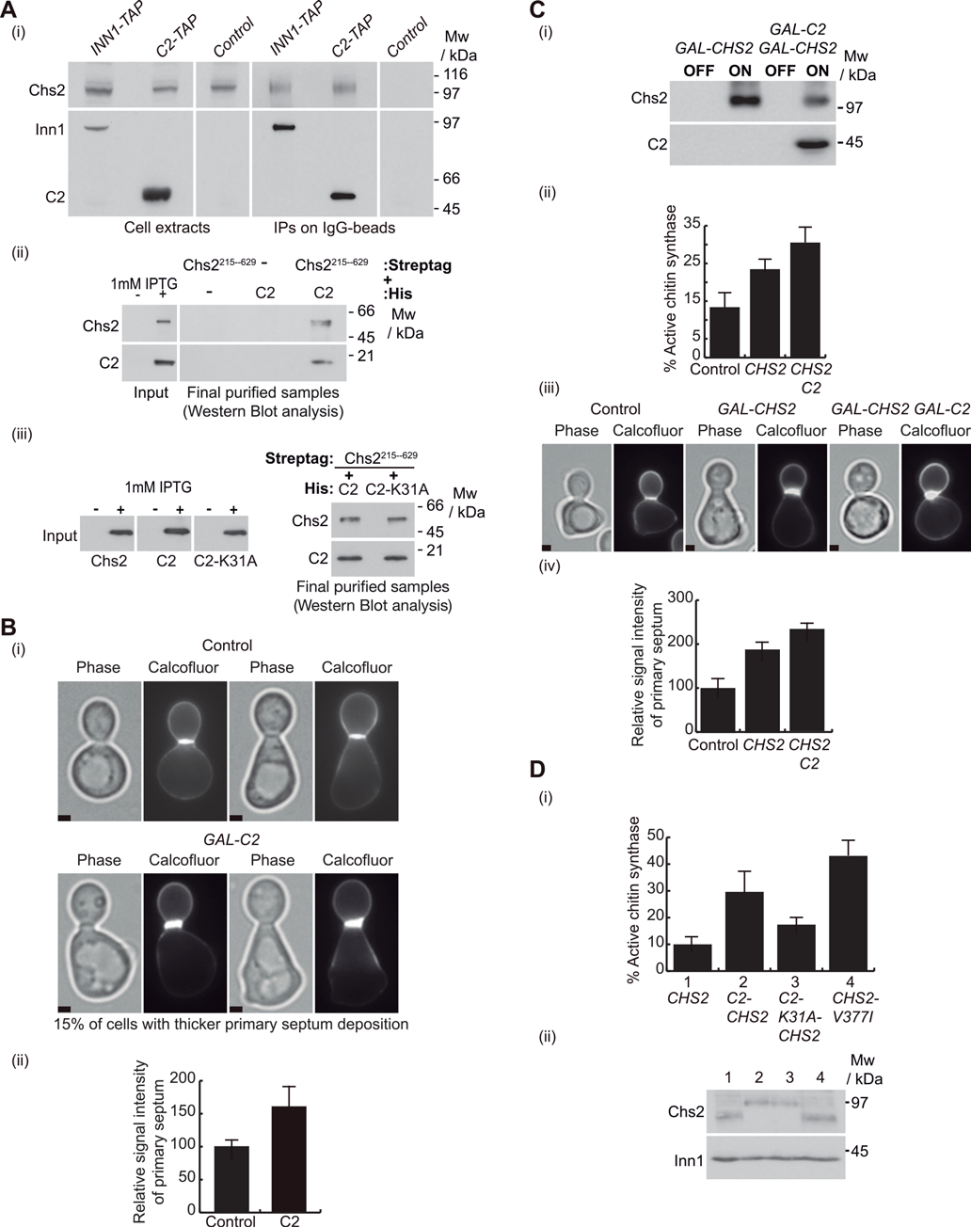
C2 domain of Inn1 directly binds to and regulates the catalytic activity of Chs2. **(A)** *INN1-TAP CHS2-9MYC* (YMF38), *C2-TAP CHS2-9MYC* (YMF88) and control (YAD382) strains were grown at 24°C in YPD medium, arrested in G1 phase by the addition of alpha factor, and then released in YPD medium for 105 minutes. Cell extracts were made and Inn1-TAP or C2-TAP were immunoprecipitated on IgG beads before detection of the indicated proteins by immunoblotting (i). After induction with IPTG, pairs of*E*. *coli* cultures expressing 6His-tagged-Inn1-C2 and Strep-tag-Chs2-215-629 were mixed and used to purify putative protein complexes following scheme in S1A (see Materials and Methods). The final purified fractions were analysed by SDS-PAGE and tagged proteins were detected with anti-Streptag or anti-His antibodies (ii). Pairs of *E*. *coli* cultures expressing 6His-tagged-Inn1-C2 or 6His-tagged-Inn1-C2-K31A were mixed with Strep-tag-Chs2-215-629 and used to purify putative protein complexes as in (ii). The final purified fractions were analysed by SDS-PAGE and tagged proteins were detected with anti-Streptag or anti-His antibodies (iii). **(B)** *chs3Δ* control (YMF505) and *GAL-C2 chs3Δ* (YRK3) cells were grown in YPRaff medium at 24°C and synchronised in G1 with alpha factor. Subsequently, cells were released in YPGal for 135 minutes from G1 block in the presence of calcofluor to visualise primary septum deposition. 100 cells with primary septum for each sample were examined and we found that 15% of *GAL-C2 chs3Δ* cells had clearly higher intensity at the primary septum than the average intensity in control cells. Examples of these cells are shown in (i). Scale bars correspond to 2μm. The relative signal intensity of primary septum was measured for 100 cells and compared to control cells, where signal intensity was set to 100% (ii). **(C)** C2 domain of Inn1 increases the catalytic activity of Chs2. The protein levels of overexpressed Chs2 and C2 proteins (i) and percentage of active chitin synthase (ii) in *chs3Δ* control cells and cells lacking Chs3 and overexpressing either *GAL-CHS2* (YMF687) or *GAL-CHS2 GAL-C2* (YMF581) were determined in membranes isolated from asynchronous cultures (see Materials and Methods). Control, *GAL-CHS2* (YMF687) and *GAL-CHS2 GAL-C2* (YMF581) were grown as in (B) and stained with calcofluor to visualise primary septum deposition. 100 cells with primary septum for each sample were examined and examples of these cells are shown in (iii) and the relative signal intensity of primary septum was measured and compared to control cells, where signal intensity was set to 100% (iv). Scale bars correspond to 2μm. **(D)** The chitin synthase activity in *chs3Δ* cells expressing *CHS2* (YMF191), *C2-CHS2* (YMF172), *C2-K31A-CHS2* (YMF174) or*CHS2-V377I* (YMF192) was determined as in (C) (i). Cells were grown in YPD containing 0.1mM CuSO_4_ since *CHS2* and *CHS2* fusions were under the control of the *CUP1* promoter and protein expression levels of Chs2 and its fusions were determined (ii). Note that *CHS2* is highly expressed in (C), under the control of the *GAL1-10* promoter, whereas *CHS2* levels are much reduced in (D), under the *CUP1* promoter control.

**Fig 4:**
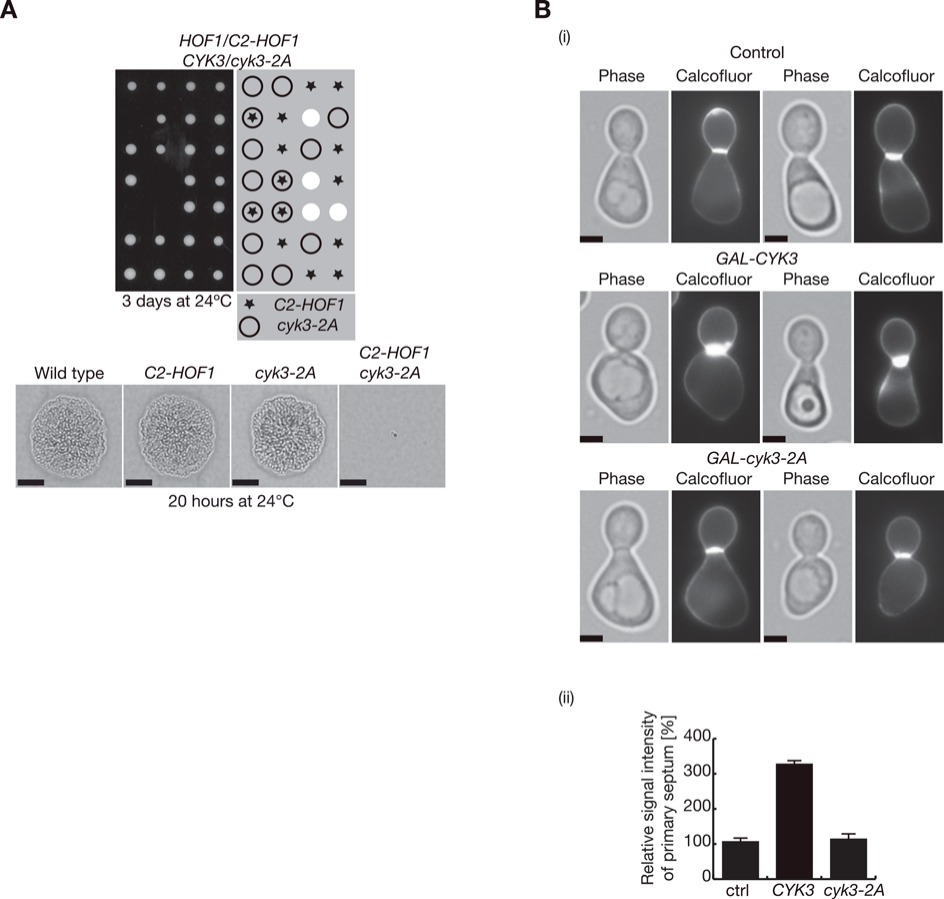
Transglutaminase-like domain of Cyk3 is important to stimulate chitin synthesis during cell division. **(A)** Tetrad analysis of the meiotic progeny from the indicated diploid strain (YMF669) shows that inactivation of the transglutaminase-like domain of Cyk3 (*cyk3-2A* allele, which contains two point mutations, D578A and H563A) is lethal when combined with *C2-HOF1*. Scale bars correspond to 20μm. **(B)** Control (YMF505), *GAL-CYK3* (YIMP235) and *GAL-cyk3-2A* (YMF576) cells were grown in YPRaff medium at 24°C, synchronised in G1 with alpha factor, and cells were released in YPGal for 135 minutes in the presence of calcofluor to visualise primary septum deposition (i). The relative signal intensity of primary septum was measured for 100 cells and compared to control cells, where signal intensity was set to 100% (ii). Scale bars correspond to 2μm.

**Fig 9:**
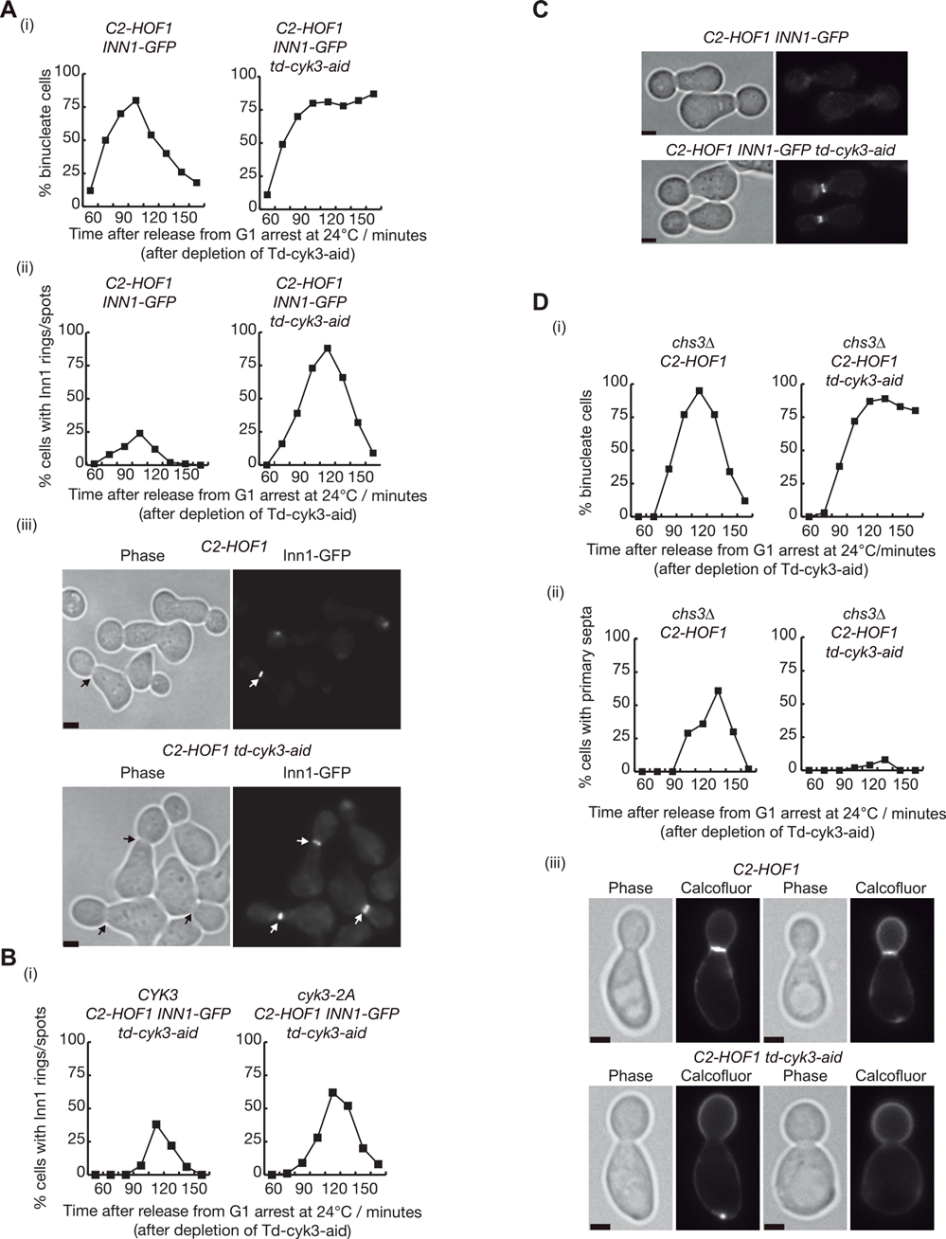
Inn1 and Cyk3 finely control primary septum deposition during cytokinesis. **(A)** The indicated strains *INN1-GFP C2-HOF1* (YIMP196) and *INN1-GFP td-cyk3-aid C2-HOF1* (YIMP198) were released from G1 arrest at 24°C in YPRaff medium and cells were allowed to progress through the cell cycle at 24°C in YPGal after depleting Td-Cyk3-aid. The proportion of binucleate cells was monitored (i) in parallel with recruitment of Inn1 to the bud-neck (ii). Examples of cells with Inn1-GFP rings at the bud-neck are shown for the 105’ time-point. Scale bars indicate 2μm (iii). **(B)** The indicated strains *INN1-GFP C2-HOF1 td-cyk3-aid CYK3* (YMF951) and *INN1-GFP C2-HOF1 td-cyk3-aid cyk3-2A* (YMF950) were grown as in (A). Recruitment of Inn1 to the bud-neck was determined. **(C)** The indicated strains from Fig 9A *INN1-GFP C2-HOF1* (YIMP196) and *INN1-GFP td-cyk3-aid C2-HOF1* (YIMP198) were released from G1 arrest at 24°C in YPRaff medium after depletion of Td-Cyk3-aid. After cells budded and completed S-phase, nocodazole was added to synchronise the cells in G2-M-phase. The recruitment of Inn1 to the bud-neck in cells arrested in G2-M phase was monitored and examples of cells with Inn1-GFP rings at the bud-neck in nocodazole-arrested cells are shown. Scale bars indicate 2μm. **(D)** Control (YIMP234) and *td-cyk3-aid C2-HOF1*(YIMP246) strains were grown as described in (A) but calcofluor was added upon release from G1 block. The proportion of binucleate cells was determined (i) and the number of cells forming primary septa stained with calcofluor was counted (ii). Examples of calcofluor-stained cells from 135 minutes after release from G1 block (iii). Scale bars correspond to 2μm.
