# Supplementary material for: Ingression Progression Complexes Control Extracellular Matrix Remodelling during Cytokinesis in Budding Yeast
Source: PLoS Genet. 2016 Feb 18;12(2):e1005864. doi: 10.1371/journal.pgen.1005864 (PMC4758748; doi:10.1371/journal.pgen.1005864)
Supplement: S1 Table — (DOC) [file pgen.1005864.s011.doc]

| **Strain Name** | **Genotype** | **Sourc*e*** |
| --- | --- | --- |
| W303-1 | *MAT***a /** *MATα*  *ade2-1 / ade2-1 ura3-1 / ura3-1 his3-11,15 / his3-11,15 trp1-1 / trp1-1 leu2-3,112 / leu2-3,112 can1-100 / can1-100* | R. Rothstein |
| W303-1a | *MAT***a** *ade2-1 ura3-1 his3-11,15 trp1-1 leu2-3,112 can1-100* | R. Rothstein |
| YMF38 | *MAT***a** *ade2-1 ura3-1 his3-11,15 trp1-1 leu2-3,112 can1-100*  *CHS2-9MYC (K.l.TRP1) INN1-TAP (kanMX) pep4∆::URA3 (URA3) ADE2* | This study |
| YMF79 | *MAT***a** *ade2-1 ura3-1 his3-11,15 trp1-1 leu2-3,112 can1-100*  *CHS2-9MYC (K.l.TRP1) leu2-3,112::TETO2-CTAP4 (LEU2) pep4∆::URA3 (URA3) ADE2* | This study |
| YMF82 | *MAT***a** *ade2-1 ura3-1 his3-11,15 trp1-1 leu2-3,112 can1-100*  *CHS2-9MYC (K.l.TRP1) leu2-3,112::pRS305-Cterminus-TAP (LEU2, TRP1) pep4∆::URA3 (URA3) ADE2* | This study |
| YMF88 | *MAT***a** *ade2-1 ura3-1 his3-11,15 trp1-1 leu2-3,112 can1-100*  *CHS2-9MYC (K.l.TRP) leu2-3,112::pRS305-C2-TAP (LEU2) pep4∆::URA3 (URA3) ADE2* | This study |
| YMF149 | *MAT***a** *ade2-1 ura3-1 his3-11,15 trp1-1 leu2-3,112 can1-100*  *IQG1-6HA (K.l.TRP) INN1-TAP (kanMX) pep4∆::URA3 (URA3) ADE2* | This study |
| YMF152 | *MAT***a** *ade2-1 ura3-1 his3-11,15 trp1-1 leu2-3,112 can1-100*  *IQG1-6HA (K.l.TRP) pep4∆::URA3 (URA3) ADE2* | This study |
| YMF172 | *MAT***a** *ade2-1 ura3-1 his3-11,15 trp1-1 leu2-3,112 can1-100*  *chs3∆::hphNT (hphNT) CUP1-C2-CHS2 (HIS3MX)* | This study |
| YMF174 | *MAT***a** *ade2-1 ura3-1 his3-11,15 trp1-1 leu2-3,112 can1-100*  *chs3∆::kanMX (kanMX) CUP1-C2-K31A-CHS2 (HIS3MX)* | This study |
| YMF191 | *MAT***a** *ade2-1 ura3-1 his3-11,15 trp1-1 leu2-3,112 can1-100*  *chs3∆::kanMX (kanMX) CUP1-CHS2 (HIS3MX)* | This study |
| YMF192 | *MAT***a** *ade2-1 ura3-1 his3-11,15 trp1-1 leu2-3,112 can1-100*  *chs3∆::kanMX (kanMX) CUP1-CHS2-V377I (HIS3MX, hphNT)* | This study |
| YMF305 | *MAT***a** *ade2-1 ura3-1 his3-11,15 trp1-1 leu2-3,112 can1-100*  *ubr1∆::GAL-HA-UBR1 (HIS3) CHS2-GFP (K.l.TRP1) iqg1-td (hphNT)* | This study |
| YMF330 | *MAT***a** *ade2-1 ura3-1 his3-11,15 trp1-1 leu2-3,112 can1-100*  *ubr1∆::GAL-HA-UBR1 (HIS3) CHS2-GFP (K.l.TRP1)* | This study |
| YMF334 | *MAT***a** *ade2-1 ura3-1 his3-11,15 trp1-1 leu2-3,112 can1-100*  *ubr1∆::GAL-HA-UBR1 (HIS3) ura3-1::GAL-O.s.TIR1-9MYC (URA3) CUP1-C2-HOF1 (HIS3MX)* | This study |
| YMF343 | *MAT***a** *ade2-1 ura3-1 his3-11,15 trp1-1 leu2-3,112 can1-100*  *ubr1∆::GAL-HA-UBR1 (HIS3) ura3-1::GAL-O.s.TIR1-9MYC (URA3) CHS2-GFP ( K.l.TRP1) td-cyk3-aid (K.l.TRP1, hphNT)* | This study |
| YMF356 | *MAT***a** *ade2-1 ura3-1 his3-11,15 trp1-1 leu2-3,112 can1-100*  *ubr1∆::GAL-HA-UBR1 (HIS3) ura3-1::GAL-O.s.TIR1-9MYC (URA3) td-cyk3-aid (K.l.TRP1, hphNT) CUP1-C2-HOF1 (HIS3MX)* | This study |
| YMF362 | *MAT***a** *ade2-1 ura3-1 his3-11,15 trp1-1 leu2-3,112 can1-100*  *HOF1-9MYC (HIS3MX) IQG1-6HA (K.l.TRP1) MYO1-5FLAG (hphNT) pep4∆::URA3 (URA3) ADE2* | This study |
| YMF373 | *MAT***a** *ade2-1 ura3-1 his3-11,15 trp1-1 leu2-3,112 can1-100*  *ubr1∆::GAL-HA-UBR1 (HIS3) ura3-1::GAL-O.s.TIR1-9MYC (URA3) INN1-GFP (kanMX)* | This study |
| YMF505 | *MAT***a** *ade2-1 ura3-1 his3-11,15 trp1-1 leu2-3,112 can1-100*  *chs3∆::URA3 (URA3)* | This study |
| YMF561 | *MAT***a** *ade2-1 ura3-1 his3-11,15 trp1-1 leu2-3,112 can1-100*  *chs3∆::URA3 (URA3) trp1-1::GAL-CHS2-6HA (kanMX. TRP1) leu2-3,112::pRS305-GAL-INN1 (LEU2)* | This study |
| YMF576 | *MAT***a** *ade2-1 ura3-1 his3-11,15 trp1-1 leu2-3,112 can1-100*  *chs3∆::URA3 (URA3) leu2-3,112::pRS305-GAL-cyk3-H563A-D578A-GFP (LEU2)* | This study |
| YMF581 | *MAT***a** *ade2-1 ura3-1 his3-11,15 trp1-1 leu2-3,112 can1-100*  *chs3∆::URA3 (URA3) trp1-1::GAL-CHS2-6HA (kanMX. TRP1) leu2-3,112::pRS305-GAL-C2-TAP (LEU2)* | This study |
| YMF610 | *MAT***a** *ade2-1 ura3-1 his3-11,15 trp1-1 leu2-3,112 can1-100*  *CHS2-GFP (K.l.TRP1) leu2-3,112::pRS305-GAL-CYK3 (LEU2)* | This study |
| YMF660 | *MAT***a** *ade2-1 ura3-1 his3-11,15 trp1-1 leu2-3,112 can1-100*  *CHS2-GFP (K.l.TRP1) leu2-3,112::pRS305-GAL-C2-TAP (LEU2)* | This study |
| YMF669 | *MAT***a /** *MATα*  *ade2-1 / ade2-1 ura3-1 / ura3-1 his3-11,15 / his3-11,15 trp1-1 / trp1-1 leu2-3,112 / leu2-3,112 can1-100 / can1-100 HOF1 / CUP1-C2-HOF1 (HIS3MX) CYK3 / cyk3-H563A-D578A-GFP (LEU2)* | This study |
| YMF673 | *MAT***a** *ade2-1 ura3-1 his3-11,15 trp1-1 leu2-3,112 can1-100*  *chs3∆::URA3 (URA3) trp1-1::GAL-CHS2-6HA (kanMX. TRP1) leu2-3,112::pRS305-GAL-C2-TAP (LEU2)* | This study |
| YMF687 | *MAT***a** *ade2-1 ura3-1 his3-11,15 trp1-1 leu2-3,112 can1-100*  *chs3∆::URA3 (URA3) trp1-1::GAL-CHS2-6HA (kanMX. TRP1)* | This study |
| YMF694 | *MAT***a** *ade2-1 ura3-1 his3-11,15 trp1-1 leu2-3,112 can1-100*  *chs3∆::URA3 (URA3) CHS2-V377I (hphNT)* | This study |
| YMF891 | *MAT***a** *ade2-1 ura3-1 his3-11,15 trp1-1 leu2-3,112 can1-100*  *chs3∆::URA3 (URA3) chs2-aid (kanMX) ura3-1::ADH1-TIR1-9MYC (URA3) pRS313 (HIS3)* | This study |
| YMF892 | *MAT***a** *ade2-1 ura3-1 his3-11,15 trp1-1 leu2-3,112 can1-100*  *chs3∆::URA3 (URA3) chs2-aid (kanMX) ura3-1::ADH1-TIR1-9MYC (URA3) pRS313-CHS2-V377I (HIS3)* | This study |
| YMF893 | *MAT***a** *ade2-1 ura3-1 his3-11,15 trp1-1 leu2-3,112 can1-100*  *chs3∆::URA3 (URA3) chs2-aid (kanMX) ura3-1::ADH1-TIR1-9MYC (URA3) pRS313-CHS2-V377I-D562A (HIS3)* | This study |
| YMF950 | *MAT***a** *ade2-1 ura3-1 his3-11,15 trp1-1 leu2-3,112 can1-100*  *ubr1∆::GAL-HA-UBR1 (HIS3) ura3-1::GAL-O.s.TIR1-9MYC (URA3) CUP1-C2-HOF1 (HIS3MX) INN1-GFP (kanMX) td-cyk3-aid (K.l.TRP1, hphNT) ) leu2-3,112::pRS305-cyk3-2A (LEU2) SPC42-EQFP (HIS3)* | This study |
| YMF951 | *MAT***a** *ade2-1 ura3-1 his3-11,15 trp1-1 leu2-3,112 can1-100*  *ubr1∆::GAL-HA-UBR1 (HIS3) ura3-1::GAL-O.s.TIR1-9MYC (URA3) CUP1-C2-HOF1 (HIS3MX) INN1-GFP (kanMX) td-cyk3-aid (K.l.TRP1, hphNT) ) leu2-3,112::pRS305-CYK3 (LEU2) SPC42-EQFP (HIS3)* | This study |
| YMF953 | *MAT***a /** *MATα*  *ade2-1 / ade2-1 ura3-1 / ura3-1 his3-11,15 / his3-11,15 trp1-1 / trp1-1 leu2-3,112 / leu2-3,112 can1-100 / can1-100 HOF1 / CUP1-TG-HOF1 (kanMX) CYK3 / cyk3∆ (hphNT) CHS2 / CUP1-C2-CHS2 (HIS3MX)* | This study |
| YMF960 | *MAT***a /** *MATα*  *ade2-1 / ade2-1 ura3-1 / ura3-1 his3-11,15 / his3-11,15 trp1-1 / trp1-1 leu2-3,112 / leu2-3,112 can1-100 / can1-100 HOF1 / CUP1-TG-HOF1 (kanMX) CYK3 / cyk3-2A (K.l.TRP1) CHS2 / CUP1-C2-CHS2 (HIS3MX)* | This study |
| YIMP11 | *MAT***a /** *MATα*  *ade2-1 / ade2-1 ura3-1 / ura3-1 his3-11,15 / his3-11,15 trp1-1 / trp1-1 leu2-3,112 / leu2-3,112 can1-100 / can1-100 HOF1 / CUP1-C2-HOF1 (HIS3MX) CYK3 / cyk3∆ (K.l.TRP1) CHS2 / CHS2-V377I (hphNT)* | This study |
| YIMP12 | *MAT***a /** *MATα*  *ade2-1 / ade2-1 ura3-1 / ura3-1 his3-11,15 / his3-11,15 trp1-1 / trp1-1 leu2-3,112 / leu2-3,112 can1-100 / can1-100 HOF1 / CUP1-C2(K31A)-HOF1 (HIS3MX) CYK3 / cyk3∆ (K.l.TRP1) CHS2 / CHS2-V377I (hphNT)* | This study |
| YIMP41 | *MAT***a** *ade2-1 ura3-1 his3-11,15 trp1-1 leu2-3,112 can1-100*  *C2-HOF1 (HIS3MX) ura3-1::ADH1-OsTIR1-9MYC (URA3) cyk3-aid (kanMX)* | This study |
| YIMP43 | *MAT***a** *ade2-1 ura3-1 his3-11,15 trp1-1 leu2-3,112 can1-100*  *C2-HOF1 (HIS3MX) ura3-1::ADH1-OsTIR1-9MYC (URA3)* | This study |
| YIMP60 | *MAT***a** *ade2-1 ura3-1 his3-11,15 trp1-1 leu2-3,112 can1-100*  *ura3-1::ADH1-OsTIR1-9MYC (URA3) cyk3-aid (kanMX)* | This study |
| YIMP142 | *MAT***a** *ade2-1 ura3-1 his3-11,15 trp1-1 leu2-3,112 can1-100*  *ura3-1:: ADH1-OsTIR1-9MYC (URA3) CUP1-C2-HOF1 (HIS3MX) td-inn1-aid (K.l.TRP1, kanMX) leu2-3,112::pRS305-INN1-K31A-GFP (LEU2) cyk3-aid (kanMX)* | This study |
| YIMP147 | *MAT***a** *ade2-1 ura3-1 his3-11,15 trp1-1 leu2-3,112 can1-100*  *ura3-1:: ADH1-OsTIR1-9MYC (URA3) td-inn1-aid (K.l.TRP1, kanMX) leu2-3,112::pRS305-INN1-K31A-GFP (LEU2)* | This study |
| YIMP149 | *MAT***a** *ade2-1 ura3-1 his3-11,15 trp1-1 leu2-3,112 can1-100*  *ura3-1:: ADH1-OsTIR1-9MYC (URA3) td-inn1-aid (K.l.TRP1, kanMX) leu2-3,112::pRS305-INN1-K31A-GFP (LEU2)* | This study |
| YIMP189 | *MAT***a** *ade2-1 ura3-1 his3-11,15 trp1-1 leu2-3,112 can1-100*  *ubr1∆::GAL-HA-UBR1 (HIS3) hof1-td (hphNT) CHS2-GFP (K.l.TRP1)* | This study |
| YIMP196 | *MAT***a** *ade2-1 ura3-1 his3-11,15 trp1-1 leu2-3,112 can1-100*  *ubr1∆::GAL-HA-UBR1 (HIS3) ura3-1::GAL-O.s.TIR1-9MYC (URA3) CUP1-C2-HOF1 (HIS3MX) INN1-GFP (kanMX)* | This study |
| YIMP198 | *MAT***a** *ade2-1 ura3-1 his3-11,15 trp1-1 leu2-3,112 can1-100*  *ubr1∆::GAL-HA-UBR1 (HIS3) ura3-1::GAL-O.s.TIR1-9MYC (URA3) CUP1-C2-HOF1 (HIS3MX) INN1-GFP (kanMX) td-cyk3-aid (K.l.TRP1, hphNT)* | This study |
| YIMP206 | *MAT***a** *ade2-1 ura3-1 his3-11,15 trp1-1 leu2-3,112 can1-100*  *ubr1∆::GAL-HA-UBR1 (HIS3) ura3-1::GAL-O.s.TIR1-9MYC (URA3) CHS2-GFP ( K.l.TRP1)* | This study |
| YIMP209 | *MAT***a** *ade2-1 ura3-1 his3-11,15 trp1-1 leu2-3,112 can1-100*  *ubr1∆::GAL-HA-UBR1 (HIS3) ura3-1::GAL-O.s.TIR1-9MYC (URA3) CUP1-C2-HOF1 (HIS3MX) MYO1-GFP ( K.l.TRP1) td-cyk3-aid (K.l.TRP1, hphNT) )* | This study |
| YIMP225 | *MAT***a** *ade2-1 ura3-1 his3-11,15 trp1-1 leu2-3,112 can1-100*  *ubr1∆::GAL-HA-UBR1 (HIS3) ura3-1::GAL-O.s.TIR1-9MYC (URA3) CUP1-C2-HOF1 (HIS3MX) MYO1-GFP ( K.l.TRP1)* | This study |
| YIMP230 | *MAT***a** *ade2-1 ura3-1 his3-11,15 trp1-1 leu2-3,112 can1-100*  *ubr1∆::GAL-HA-UBR1 (HIS3) hof1-td (hphNT) inn1-td (kanMX) CHS2-GFP (K.l.TRP1)* | This study |
| YIMP234 | *MAT***a** *ade2-1 ura3-1 his3-11,15 trp1-1 leu2-3,112 can1-100*  *ubr1∆::GAL-HA-UBR1 (HIS3) ura3-1::GAL-O.s.TIR1-9MYC (URA3) CUP1-C2-HOF1 (HIS3MX) chs3∆::URA3 (URA3)* | This study |
| YIMP235 | *MAT***a** *ade2-1 ura3-1 his3-11,15 trp1-1 leu2-3,112 can1-100*  *chs3∆::URA3 (URA3) leu2-3,112::pRS305-GAL-CYK3 (LEU2)* | This study |
| YIMP240 | *MAT***a** *ade2-1 ura3-1 his3-11,15 trp1-1 leu2-3,112 can1-100*  *ura3-1:: ADH1-OsTIR1-9MYC (URA3) CUP1-C2-HOF1 (HIS3MX) td-inn1-aid (K.l.TRP1, kanMX) cyk3-aid (kanMX)* | This study |
| YIMP242 | *MAT***a** *ade2-1 ura3-1 his3-11,15 trp1-1 leu2-3,112 can1-100*  *ura3-1:: ADH1-OsTIR1-9MYC (URA3) CUP1-C2-HOF1 (HIS3MX) td-inn1-aid (K.l.TRP1, kanMX)* | This study |
| YIMP246 | *MAT***a** *ade2-1 ura3-1 his3-11,15 trp1-1 leu2-3,112 can1-100*  *ubr1∆::GAL-HA-UBR1 (HIS3) ura3-1::GAL-O.s.TIR1-9MYC (URA3) CUP1-C2-HOF1 (HIS3MX) chs3∆::URA3 (URA3) td-cyk3-aid (K.l.TRP1, hphNT)* | This study |
| YIMP247 | *MAT***a** *ade2-1 ura3-1 his3-11,15 trp1-1 leu2-3,112 can1-100*  *chs3∆::URA3 (URA3) ubr1∆::GAL-HA-UBR1 (HIS3) ura3-1::GAL-O.s.TIR1-9MYC (URA3) td-cyk3-aid (hphNT, K.l.TRP1)* | This study |
| YIMP248 | *MAT***a** *ade2-1 ura3-1 his3-11,15 trp1-1 leu2-3,112 can1-100*  *chs3∆::URA3 (URA3) ubr1∆::GAL-HA-UBR1 (HIS3) ura3-1::GAL-O.s.TIR1-9MYC (URA3)* | This study |
| YIMP253 | *MAT***a** *ade2-1 ura3-1 his3-11,15 trp1-1 leu2-3,112 can1-100*  *ubr1∆::GAL-HA-UBR1 (HIS3) CHS2-V377I-9MYC (K.l.TRP1)* | This study |
| YIMP254 | *MAT***a** *ade2-1 ura3-1 his3-11,15 trp1-1 leu2-3,112 can1-100*  *ubr1∆::GAL-HA-UBR1 (HIS3) CHS2-V377I-9MYC (K.l.TRP1)*  *hof1-td (hphNT)* | This study |
| YIMP255 | *MAT***a /** *MATα*  *ade2-1 / ade2-1 ura3-1 / ura3-1 his3-11,15 / his3-11,15 trp1-1 / trp1-1 leu2-3,112 / leu2-3,112 can1-100 / can1-100 leu2-3,112 / leu2-3,112::pRS305-GAL-CYK3 (LEU2) CHS2 / chs2∆* | This study |
| YIMP265 | *MAT***a** *ade2-1 ura3-1 his3-11,15 trp1-1 leu2-3,112 can1-100*  *ura3-1::ADH1-OsTIR1-9MYC (URA3) chs2-aid (kanMX) leu2-3,112::pRS305-GAL-CYK3-GFP (LEU2)* | This study |
| YIMP267 | *MAT***a** *ade2-1 ura3-1 his3-11,15 trp1-1 leu2-3,112 can1-100*  *ura3-1::ADH1-OsTIR1-9MYC (URA3) leu2-3,112::pRS305-GAL-CYK3-GFP (LEU2)* | This study |
| YIMP272 | *MAT***a** *ade2-1 ura3-1 his3-11,15 trp1-1 leu2-3,112 can1-100*  *ubr1∆::GAL-HA-UBR1 (HIS3) hof1-td (hphNT) trp1-1::GAL-CHS2-6HA (kanMX. K.l.TRP1)* | This study |
| YIMP273 | *MATα* *ade2-1 ura3-1 his3-11,15 trp1-1 leu2-3,112 can1-100*  *ubr1∆::GAL-HA-UBR1 (HIS3) trp1-1::GAL-CHS2-6HA (kanMX. K.l.TRP1)* | This study |
| YIMP310 | *MAT***a** *ade2-1 ura3-1 his3-11,15 trp1-1 leu2-3,112 can1-100*  *ura3-1:: ADH1-OsTIR1-9MYC (URA3) CUP1-C2-HOF1 (HIS3MX) td-inn1-aid (K.l.TRP1, kanMX) cyk3-aid (kanMX) CHS2-V377I (hphNT)* | This study |
| YIMP324 | *MATα* *ade2-1 ura3-1 his3-11,15 trp1-1 leu2-3,112 can1-100*  *ura3-1:: ADH1-OsTIR1-9MYC (URA3) td-inn1-aid (K.l.TRP1, kanMX)* | This study |
| YIMP329 | *MAT***a** *ade2-1 ura3-1 his3-11,15 trp1-1 leu2-3,112 can1-100*  *ura3-1:: ADH1-OsTIR1-9MYC (URA3) CUP1-C2-HOF1 (HIS3MX) cyk3-aid (kanMX) CHS2-V377I (hphNT)* | This study |
| YIMP334 | *MATα* *ade2-1 ura3-1 his3-11,15 trp1-1 leu2-3,112 can1-100*  *ura3-1:: ADH1-OsTIR1-9MYC (URA3) CUP1-C2-HOF1 (HIS3MX) CHS2-V377I (hphNT)* | This study |
| YIMP388 | *MAT***a /** *MATα*  *ade2-1 / ade2-1 ura3-1 / ura3-1 his3-11,15 / his3-11,15 trp1-1 / trp1-1 leu2-3,112 / leu2-3,112 can1-100 / can1-100 INN1 / inn1∆ (K.l.TRP1) HOF1 / CUP1-C2-HOF1 (HIS3MX) CYK3 / cyk3∆ (hphNT) CHS2 / CHS2-V377I (kanMX)* | This study |
| YIMP423 | *MAT***a** *ade2-1 ura3-1 his3-11,15 trp1-1 leu2-3,112 can1-100*  *CHS2-GFP (K.l.TRP1) leu2-3,112::pRS305-GAL-cyk3-2A (LEU2)* | This study |
| YIMP428 | *MAT***a /** *MATα*  *ade2-1 / ade2-1 ura3-1 / ura3-1 his3-11,15 / his3-11,15 trp1-1 / trp1-1 leu2-3,112 / leu2-3,112 can1-100 / can1-100) HOF1 / CUP1-C2-HOF1 (HIS3MX) CHS2 / CHS2-GFP (K.l.TRP1) CYK3 / td-cyk3-aid (K.l.TRP, hphNT))* | This study |
| YIMP437 | *MAT***a /** *MATα*  *ade2-1 / ade2-1 ura3-1 / ura3-1 his3-11,15 / his3-11,15 trp1-1 / trp1-1 leu2-3,112 / leu2-3,112 can1-100 / can1-100 INN1 / inn1∆ (K.l.TRP1) HOF1 / CUP1-C2-HOF1 (HIS3MX) CYK3 / cyk3-2A (hphNT) CHS2 / CHS2-V377I (kanMX)* | This study |
| YIMP452 | *MAT***a** *ade2-1 ura3-1 his3-11,15 trp1-1 leu2-3,112 can1-100*  *ubr1∆::GAL-HA-UBR1 (HIS3) ura3-1::GAL-O.s.TIR1-9MYC (URA3) CUP1-C2-HOF1 (HIS3MX) MYO1-GFP ( K.l.TRP1) SPC42-EQFP (HIS3)* | This study |
| YASD550 | *MAT***a** *ade2-1 ura3-1 his3-11,15 trp1-1 leu2-3,112 can1-100*  *ubr1∆::GAL-HA-UBR1 (HIS3) iqg1-td (hphNT) HOF1-GFP (K.l.TRP1)* | A. Sanchez-Diaz |
| YASD556 | *MAT***a** *ade2-1 ura3-1 his3-11,15 trp1-1 leu2-3,112 can1-100*  *ubr1∆::GAL-HA-UBR1 (HIS3) HOF1-GFP (K.l.TRP1)* | A. Sanchez-Diaz |
| YASD641 | *MAT***a** *ade2-1 ura3-1 his3-11,15 trp1-1 leu2-3,112 can1-100*  *CYK3-GFP (K.l.TRP1)* | A. Sanchez-Diaz |
| YASD681 | *MAT***a** *ade2-1 ura3-1 his3-11,15 trp1-1 leu2-3,112 can1-100*  *ubr1∆::GAL-HA-UBR1 (HIS3) hof1-td (hphNT)* | A. Sanchez-Diaz |
| YASD689 | *MAT***a** *ade2-1 ura3-1 his3-11,15 trp1-1 leu2-3,112 can1-100*  *ubr1∆::GAL-HA-UBR1 (HIS3) inn1-td (kanMX) CHS2-GFP (K.l.TRP1)* | A. Sanchez-Diaz |
| YASD819 | *MAT***a** *ade2-1 ura3-1 his3-11,15 trp1-1 leu2-3,112 can1-100*  *CHS2-GFP (K.l.TRP1)* | A. Sanchez-Diaz |
| YAD382 | *MAT***a** *ade2-1 ura3-1 his3-11,15 trp1-1 leu2-3,112 can1-100*  *CHS2-9MYC (K.l.TRP1) pep4∆::URA3 (URA3) ADE2* | A. Devrekanli |
| YAD394 | *MATα* *ade2-1 ura3-1 his3-11,15 trp1-1 leu2-3,112 can1-100*  *ura3-1::ADH1-OsTIR1-9MYC (URA3) chs2-aid (kanMX)* | A. Devrekanli |
| YJW15 | *MAT***a** *ade2-1 ura3-1 his3-11,15 trp1-1 leu2-3,112 can1-100*  *ura3-1:: ADH1-OsTIR1-9MYC (URA3)* | M. Kanemaki |
| YRK3 | *MAT***a** *ade2-1 ura3-1 his3-11,15 trp1-1 leu2-3,112 can1-100*  *chs3∆::URA3 (URA3) leu2-3,112::pRS305-GAL-C2-TAP (LEU2)* | This study |
