# Supplementary material for: Ingression Progression Complexes Control Extracellular Matrix Remodelling during Cytokinesis in Budding Yeast
Source: PLoS Genet. 2016 Feb 18;12(2):e1005864. doi: 10.1371/journal.pgen.1005864 (PMC4758748; doi:10.1371/journal.pgen.1005864)
Supplement: S2 Table — (DOC) [file pgen.1005864.s012.doc]

| **Plasmid** | **Expressed proteins** |
| --- | --- |
| pMF36 | Streptag-Chs2-215-629 |
| pMF15 | Streptag-Chs2-1-629 |
| pASD1 | 6His-Inn1 |
| pMF49 | Streptag-Chs2-V377I-215-629 |
| pMF52 | 6His-Inn1-K31A |
| pAD12 | 6His-C2 |
| pMF103 | 6His-C2-K31A |
| pMF22 | 6HiS-Cyk3 |
| pMF61 | Cyk3 |
